# Association of plasma ferritin and plasma iron at time of vaccination with the immune response to SARS-CoV-2 vaccination: a longitudinal cohort study

**DOI:** 10.3389/fimmu.2026.1764884

**Published:** 2026-03-04

**Authors:** Giulia Pestoni, Dominik Menges, Craig Fenwick, Pornpimol Scheuchzer, Julia Braun, Sarah R. Haile, Tala Ballouz, Christophe Zeder, Nicole U. Stoffel, Michael B. Zimmermann, Milo A. Puhan, Anja Frei, Diego Moretti

**Affiliations:** 1 Nutrition Group, Swiss Distance University of Applied Sciences (FFHS)/University of Applied Sciences and Arts of Southern Switzerland (SUPSI), Zurich, Switzerland; 2 Department of Epidemiology, Epidemiology, Biostatistics and Prevention Institute (EBPI), University of Zurich (UZH), Zurich, Switzerland; 3 Service of Immunology and Allergy, Lausanne University Hospital (CHUV), University of Lausanne (UNIL), Lausanne, Switzerland; 4 Department of Chemistry and Applied Biosciences, Institute of Pharmaceutical Sciences, ETH Zurich, Zurich, Switzerland; 5 Department of Health Sciences and Technology, Institute of Food, Nutrition and Health, ETH Zurich, Zurich, Switzerland; 6 Nuffield Department of Orthopedics, Rheumatology and Musculoskeletal Medicine, Botnar Research Centre, University of Oxford, Oxford, United Kingdom; 7 Medical Research Council Translational Immune Discovery Unit, MRC Weatherall Institute of Molecular Medicine, John Radcliffe Hospital, University of Oxford, Oxford, United Kingdom

**Keywords:** antibody response, iron status, neutralizing antibody response, plasma ferritin, plasma iron, SARS-CoV-2, vaccination

## Abstract

**Introduction:**

Recent studies have shown a link between iron status and immune response following infection or vaccination. We aimed to investigate whether plasma ferritin and plasma iron concentrations at time of vaccination were associated with the development and temporal decay of immune response to SARS-CoV-2 vaccination over 6 months.

**Materials and methods:**

We used data from the Zurich SARS-CoV-2 Vaccine Cohort (n=572). Participants were recruited from a random sample stratified by age groups (18–64 years, >65 years) and vaccine types (Pfizer-BioNTech BNT162b2, Moderna mRNA-1273, Johnson & Johnson JNJ-78436735). Iron parameters were measured at baseline (prior to vaccination), whereas different immunity markers were measured at baseline, 4 weeks, 6 weeks, 3 months, and 6 months. We investigated the association between plasma ferritin and plasma iron levels and immunity markers using linear mixed-effect models, and estimated half-life based on linear decay models.

**Results:**

Plasma ferritin and plasma iron concentrations were within the normal physiological range, and the prevalence of iron deficiency (4.5%) and inflammation (2.3%) was low. For every 50 μg/L increase in plasma ferritin concentration, we observed a 5.2% increase in Anti-S IgG antibodies, and a 13.6-19.9% increase in neutralizing antibodies against the Ancestral, Delta, and Omicron BA1 viral variants. Similarly, the highest plasma ferritin quartile showed a 14.9% increase in Anti-S IgG antibodies, and a 47.1-82.2% increase in Anti-Ancestral, Anti-Delta, and Anti-Omicron neutralizing antibodies compared to the lowest quartile. Despite high concentrations at 6 months, shorter mean half-lives of Anti-S IgG antibodies were observed in the highest quartiles of plasma ferritin concentrations (Q3: 121.4 days; Q4: 109.8 days *vs*. Q1: 152.1 days). Plasma iron results were less consistent and generally no evidence for associations was found.

**Conclusion:**

In this predominantly iron-replete cohort, higher plasma ferritin at the time of vaccination was associated with stronger vaccination-induced humoral immune responses to SARS-CoV-2 over 6 months.

## 1 Introduction

The COVID-19 global pandemic, caused by the severe acute respiratory syndrome coronavirus 2 (SARS-CoV-2), is estimated to have resulted in over 700 million people diagnosed, and over 7 million deaths by the end of 2024 (1). Vaccination is considered the most effective mitigation strategy to reduce disease severity during acute infection. Consequently, countries worldwide invested substantial resources into ensuring a rapid roll-out of SARS-CoV-2 vaccines and promoting their uptake within the population.

Iron deficiency remains a prominent nutritional deficiency and is one of the leading causes of years lived with disability worldwide (2). In Western Europe, it has been estimated that 40-55% of women of reproductive age have depleted iron stores, with 10-30% having iron deficiency (3). Iron deficiency can be divided into absolute and functional iron deficiency, the latter being characterized by hypoferremia and iron maldistribution, which can equally result in tissue and erythroid deficiency in a similar manner to absolute iron deficiency (4).

Iron status has been linked to the development of innate and acquired immunity in several studies. T cells from iron-deficient elderly individuals showed less proliferative capacity upon ex vivo stimulation with mitogens than those from iron-replete controls (5). Similarly, mitogenic stimulation of peripheral blood mononuclear cells (PBMCs) from iron-deficient children showed reduced IL-2 production (6, 7). Conversely, iron supplementation has been shown to increase the number of total and mature T cells in iron-deficient children (7). Jabara et al. (8) reported that children presenting a mutation in the encoding region for transferrin receptor 1 (TfR1), which impairs iron acquisition, have severe immunodeficiency associated with reduced memory B cells, inhibited T and B cell proliferation ex vivo, and antibody class-switching, resulting in severe and sometimes fatal infections (8).

Recent studies suggest that iron deficiency also impairs immune response following vaccination (9–11). In a randomized controlled trial in Kenyan infants, Stoffel et al. showed higher anti-measles IgG concentration and avidity in children receiving iron supplementation at time of vaccination (9). In a birth cohort of Kenyan newborns followed for 18 months, high hemoglobin at time of vaccination was associated with higher anti-diphtheria IgG, anti-pertussis IgG, and anti-pneumococcus serotype 19 IgG antibodies. Moderate to severe anemia and soluble transferrin receptor (sTfR) at time of vaccination were both risk factors for seronegativity against diphtheria and pneumococcus serotype 19 (9). In another study, patients with severe hypoferremia associated with a rare form of iron refractory anemia due to a mutation in the TMPRSS6 hepcidin regulation gene showed a lower vaccine-inducible antibody response than control individuals (10). Similarly, hypoferremia has been associated with a lower antibody response to measles vaccines compared to the antibody response in iron-sufficient controls (11).

Shortly after SARS-CoV-2 vaccines became available, a working group of the European Hematology Association issued an expert recommendation, advising to correct iron deficiency in individuals with hematologic conditions before the administration of the SARS-CoV-2 vaccines (12, 13). However, SARS-CoV-2 vaccines are remarkably protective even in populations where iron deficiency may be prevalent (14). This underscores the need for further research, particularly in exploring the immune response in more detail, including the response to more resistant variants, and characterizing speed and duration of the immune response after administration of SARS-CoV-2 vaccines (12). These have not been investigated systematically to date (13). Therefore, using data from a population-based, prospective cohort of SARS-CoV-2 vaccinated individuals, we aimed to investigate whether plasma ferritin and plasma iron concentrations at time of vaccination were associated with the development and temporal decay of markers of immune response to SARS-CoV-2 vaccination over 6 months.

## 2 Materials and methods

### 2.1 Design and study population

The analyses were conducted using data and samples from the population-based, prospective Zurich SARS-CoV-2 Vaccine Cohort study (15). Participants were recruited from a random sample of individuals receiving a SARS-CoV-2 vaccine at the reference vaccination center of the Canton of Zurich in Switzerland (Corona Center of the University of Zurich). The study sample was stratified by two age groups (18–64 years, 65 years and older) and by the three vaccine types available in Switzerland during the recruitment period (Pfizer-BioNTech BNT162b2, Moderna mRNA-1273, Johnson & Johnson JNJ-78436735). The vaccines were approved by the Swiss Agency for Therapeutic Products between December 2020 and March 2021 (16). Recruitment of study participants took place between March and July 2021 for individuals receiving an mRNA vaccine (BNT162b2 or mRNA-1273), and between October 2021 and January 2022 for individuals receiving the JNJ-78436735 adenovirus viral vector vaccine. Individuals vaccinated with an mRNA vaccine received two vaccine doses approximately 4 weeks apart. Individuals vaccinated with the JNJ-78436735 vaccine received one vaccine dose. The present study included up to 6 months of follow-up. While cohort follow-up continued beyond this time period, we did not include later timepoints in this study in light of our primary research question and to minimize the influence of booster vaccinations and breakthrough infections due to the Omicron SARS-CoV-2 variant arriving in Switzerland in December 2021.

Exclusion criteria for participation in the Zurich SARS-CoV-2 Vaccine Cohort study were: younger than 18 years, unable to follow study procedures, insufficient knowledge of the German language, prior SARS-CoV-2 vaccination in another center, and primary residence outside of the Canton of Zurich. Additionally, to increase representativeness, individuals registering as belonging to one of the following groups at the beginning of the vaccination campaign were excluded, since they represent specific population groups with priority access to vaccination: health care workers, caretakers of high-risk individuals, individuals living in communal facilities, and individuals with high-risk diseases (i.e., advanced stages of diseases such as decompensated heart or liver failure). If these individuals were registered as belonging to a regular priority group, they were eligible for inclusion. Additionally, for this analysis, participants were excluded if blood samples for iron status measurements were not available. The final study population included in the present analyses consisted of 572 individuals. A participant flow diagram is presented in **Supplementary Figure S1**.

The Research Electronic Data Capture platform (REDCap) was used for data collection. All procedures were approved by the ethics committee of the Canton of Zurich (BASEC 2021-00273) and written informed consent was obtained from all participants. The study was prospectively registered at the ISRCTN registry (ISRCTN 15499304).

### 2.2 Self-reported information

Participants were asked to complete a self-administered electronic questionnaire at baseline and at 4 weeks, 6 weeks, and approximately 3 months and 6 months after baseline. The questionnaire included questions on sociodemographic factors, smoking history, medical history, presence of comorbidities (i.e., hypertension, diabetes, cardiovascular disease, chronic respiratory disease, chronic kidney disease, cancer, or immunosuppression), SARS-CoV-2 exposure- and infection-related information, and vaccination-related information.

### 2.3 Collection and isolation of plasma

Collection of peripheral venous blood samples was performed by trained personnel at baseline and at 4 weeks, 6 weeks, 3 months, and 6 months after baseline. The timing of blood collection was not standardized in the Zurich SARS-CoV-2 Vaccine Cohort Study. During the baseline and the 4 weeks visit (for participants receiving two doses of an mRNA vaccine), blood samples were collected immediately before vaccination. Blood samples were collected in K2-EDTA vacutainer tubes and subsequently centrifuged to obtain plasma. Plasma aliquots were then frozen and stored until further analyses.

### 2.4 Measurements of iron parameters

Plasma ferritin, sTfR, retinol-binding protein (RBP), high-sensitive C-reactive protein (CRP) and alpha(1)-acid glycoprotein (AGP) were measured in the baseline samples using a combined sandwich enzyme-linked immunosorbent assay (17). Iron deficiency was defined as ferritin < 25 μg/L (18, 19). Risk of iron overload was defined as ferritin > 150 μg/L for women of reproductive age and ferritin > 200 μg/L for men and post-menopausal women, according to guidelines of the World Health Organization (20). Total body iron stores were calculated based on sTfR and ferritin using the method by Cook at al (21). Systemic inflammation was defined as CRP ≥ 5 mg/L or AGP ≥ 1 g/L.

Plasma iron was measured in the baseline samples in duplicates by inductively coupled plasma-mass spectrometry (ICP-MS; Q-ICP-MS iCap RQ, Thermo Scientific). Plasma iron concentrations were corrected for hemoglobin iron potentially present in the samples as consequence of hemolysis. The degree of hemolysis in plasma samples was determined by spectrophotometric measurement at 414 nm and compared to a calibration curve prepared from plasma and hemolyzed whole blood (22). Hemolysis was additionally checked visually using a color scale. Plasma samples were excluded when too little volume was available for measurements (n=2), when plasma iron values were implausibly high due to hemolysis (n=5), when correction for hemoglobin was impossible (n=1), and when the discrepancy between the duplicate plasma iron measurements was > 40% (n=2). This resulted in a study sample of n=562 individuals for plasma iron analyses (**Supplementary Figure S1**).

### 2.5 Measurements of spike-specific IgA and IgG antibodies and neutralization assays

Spike (S)-specific IgA and IgG antibody measurements were performed at baseline, and at 4 weeks, 6 weeks, 3 months, and 6 months after baseline. Plasma samples were thawed, and levels of S-specific IgA and IgG were measured by Luminex assay. Measurement procedures were described in detail elsewhere (23, 24). Antibody levels were reported as mean fluorescence intensity (MFI) ratios, which was calculated as the MFI in the sample divided by the MFI in seronegative control samples. The lower limit of MFI ratios was restricted to 1, representing equal fluorescence intensity as negative control samples. Seropositivity was defined as MFI ratios of > 6.5 for IgA and > 6.0 for IgG (24).

Measurements of neutralizing antibodies (NAb) were performed at 4 weeks, 3 months, and 6 months after baseline in a subsample of the study population (n=214). Frozen plasma samples were thawed, and the presence of SARS-CoV-2 NAb was assessed using a virus-and cell-free, Luminex-based assay described in detail elsewhere (23, 25). Based on the measurements, a half maximal inhibitory concentration serum dilution (IC_50_) was calculated, with an IC_50_ of 50 determined as a specificity cutoff to minimize the detection of false-positive samples.

### 2.6 Statistical analysis

Descriptive statistics were used to describe the study population. Continuous variables were reported as mean (standard deviation, SD) or as geometric mean (95% confidence interval, CI) when not normally distributed.

To investigate the longitudinal association between plasma ferritin or plasma iron levels at time of vaccination and antibody or NAb levels as markers of immune response, linear mixed-effect models were fitted with a random intercept for individuals. Iron parameters were included either as continuous variables or participants were divided into quartiles according to concentrations of iron parameters at time of vaccination. Models including plasma ferritin were adjusted for antibody levels at baseline, vaccine type and number of vaccine doses received, time point of study visit, re-exposure at study visit (defined as a diagnosed SARS-CoV-2 infection or subsequent additional vaccination), CRP, AGP, RBP, age, sex, and smoking status. The timing of blood collection across quartiles of plasma iron was balanced (Q1: 11:59, Q2: 12:11, Q3: 12:26, Q4: 12:07). Despite this, models including plasma iron were further adjusted for time of the day of study visit (as numeric time), as plasma iron concentrations show circadian variations (26).

We also investigated the association of plasma ferritin and plasma iron levels with antibody or NAb levels at single time points of study visits (i.e., at 4 weeks, 6 weeks, 3 months, and 6 months for Anti-S IgA and Anti-S IgG; at 4 weeks, 3 months, and 6 months for Anti-Ancestral, Anti-Delta, and Anti-Omicron BA1 viral variant NAb). Models including plasma ferritin were adjusted for antibody levels at baseline, vaccine type and number of vaccine doses received, re-exposure at corresponding time point of study visit, CRP, AGP, RBP, age, sex, and smoking status. Models including plasma iron were further adjusted for time of the day of study visit.

Participants with missing information on smoking status and missing data points on immunity markers at any time point were excluded from statistical modeling, and all immunity markers were logarithmically transformed using log10. When included as continuous variable in the models, plasma ferritin was rescaled to reflect changes in immunity markers per 50 μg/L increase. The original scale was used for plasma iron, reflecting changes in immunity markers per 1 μg/mL increase. Previous studies on influenza vaccines have reported that antibody levels prior to vaccination affect the post-vaccination immune response (27). Therefore, we adjusted our models for baseline antibody levels. Baseline Anti-S IgA level was used in models investigating Anti-S IgA, whereas baseline Anti-S IgG level was used in models investigating Anti-S IgG. Since no NAb measurements were performed at baseline, the baseline Anti-S IgG level was used to adjust models investigating Anti-Ancestral, Anti-Delta, and Anti-Omicron NAb, as this was considered the most robust measure of the strength of the immune response.

As sensitivity analyses, all models were fitted by adding comorbidities as adjusting factors, by excluding participants with inflammation, and by excluding CRP and AGP as adjusting factors. In addition, analyses of NAb were adjusted for seropositivity at baseline and knowledge of a prior infection, rather than for baseline Anti-S IgG level.

To estimate decay times, we followed the procedure described by Menges et al. (23). The maximum antibody concentration defined as the maximum antibody level between week 4, week 6, and month 3, was determined for each participant. The data were then restricted to the corresponding time point and all subsequent time points, with the time axis rescaled to the time since maximum antibody concentration so that the data represented a descending slope of antibody levels (decay curve). In these analyses, participants never testing positive for the respective immunity marker were excluded. Additionally, time points in which participants reported a re-exposure as well as time points in which antibody levels increased (post-vaccination or potential undiagnosed SARS-CoV-2 infection) were also excluded. To investigate the decay of immunity markers by plasma ferritin and plasma iron levels at time of vaccination, univariable and multivariable linear mixed-effect models with a random intercept for individuals were fitted. Univariable linear mixed-effect models included an interaction term between days since maximum antibody concentration and plasma ferritin or plasma iron quartiles. Multivariable linear mixed effect models were further adjusted for maximum antibody concentration, vaccine type and number of vaccine doses received, CRP, AGP, RBP, age, sex, and smoking status for plasma ferritin and additionally adjusted for time of the day of study visit for plasma iron. All immunity markers were logarithmically transformed using the natural logarithm. The half-life in days was then calculated using the following formula:

       Half-life = log (0.5)/β

where β is the model-derived estimate of the interaction term between days since maximum antibody concentration and plasma ferritin or plasma iron quartiles. 95% CI were calculated using the delta method and p-values derived with t-statistics using the Satterthwaite’s approximation method for degrees of freedom. Half-life for Anti-Omicron NAb could not be estimated.

Statistical analyses were performed using the R software (version 4.4.1 for Windows). The following packages were used: *car, data.table, DescTools, dplyr, ggplot2, gridExtra, hms, lme4, lmerTest, msm, plyr, string*, and *tidyr*. A p-value of < 0.05 was considered as statistically significant in all analyses.

## 3 Results

Out of 572 participants, 44.1% were male (**Table 1**). The mean age was 56.0 years (SD 18.1), and the mean body mass index (BMI) was 24.2 kg/m^2^ (SD 3.9). More than half of the participants were non-smokers (56.5%) and almost one third had at least one reported comorbidity (27.6%). Most participants received 2 doses of an mRNA vaccine (Pfizer-BioNTech BNT162b2 or Moderna mRNA-1273, 68.2%). Seropositivity at baseline (defined as MFI ratio > 6.5 for IgA or MFI ratio > 6.0 for IgG antibodies), was 11.5% in the overall study population, and 8.3%, 10.4% and 17.0%, respectively, in participants vaccinated with BNT162b2, mRNA-1273, and JNJ-78436735. Re-exposure at 6 months was 12.6% in the overall study population, and 1.9%, 3.5% and 37.0% respectively in participants vaccinated with BNT162b2, mRNA-1273, JNJ-78436735.

**Table 1:** Characteristics and antibody responses of participants of the Zurich SARS-CoV-2 vaccine cohort study overall and by sex and age group (n = 572).

Participants' characteristics	Overall	Sex		Age group	
		Males	Females	18–64 years	65+ years
	n = 572	n = 252	n = 320	n = 307	n = 265
Sex, n (%)					
Male	252 (44.1)	252 (100)	0 (0.0)	145 (47.2)	107 (40.4)
Age (years)	56.0 (18.1)	55.3 (18.3)	56.6 (17.9)	41.7 (11.6)	72.6 (6.5)
BMI (kg/m^2^)	24.2 (3.9)	25.0 (3.5)	23.5 (4.1)	23.8 (3.7)	24.6 (4.1)
Smoking status, n (%)					
Non-smoker	323 (56.5)	136 (54.0)	187 (58.4)	176 (57.3)	147 (55.5)
Ex-smoker	138 (24.1)	70 (27.8)	68 (21.2)	61 (19.9)	77 (29.1)
Smoker	103 (18.0)	43 (17.1)	60 (18.8)	66 (21.5)	37 (14.0)
Missing	8 (1.4)	3 (1.2)	5 (1.6)	4 (1.3)	4 (1.5)
Comorbidities, n (%)	158 (27.6)	73 (29.0)	85 (26.6)	22 (7.2)	136 (51.3)
Vaccine type and dose, n (%)					
BNT162b2–2 doses	202 (35.3)	91 (36.1)	111 (34.7)	102 (33.2)	100 (37.7)
mRNA-1273–2 doses	188 (32.9)	79 (31.3)	109 (34.1)	89 (29.0)	99 (37.4)
BNT162b2/mRNA-1273–1 dose	17 (3.0)	5 (2.0)	12 (3.8)	13 (4.2)	4 (1.5)
JNJ-78436735–1 dose	165 (28.8)	77 (30.6)	88 (27.5)	103 (33.6)	62 (23.4)
Anti-S IgA at baseline					
MFI ratio	1.5 (1.4-1.6)	1.5 (1.5-1.5)	1.5 (1.5-1.5)	1.5 (1.5-1.5)	1.4 (1.4-1.4)
Positive, n (%)	46 (8.0)	19 (7.5)	27 (8.4)	27 (8.8)	19 (7.2)
Anti-S IgG at baseline					
MFI ratio	1.4 (1.3-1.6)	1.5 (1.5-1.5)	1.4 (1.4-1.4)	1.6 (1.6-1.6)	1.3 (1.3-1.3)
Positive, n (%)	52 (9.1)	26 (10.3)	26 (8.1)	35 (11.4)	17 (6.4)
Re-exposure at 6 months, n (%)	72 (12.6)	32 (12.7)	40 (12.5)	54 (17.6)	18 (6.8)

Continuous variables are given as mean (SD) or geometric mean (95% CI), categorical variables as n (%).

Comorbidities included hypertension, diabetes, cardiovascular disease, respiratory disease, chronic kidney disease, cancer, and immunosuppression.

BMI, body mass index; CI, confidence interval; MFI, mean fluorescence intensity; NAb, neutralizing antibodies; SD, standard deviation.

Plasma ferritin concentrations (geometric mean [95% CI]: 88.1 μg/L [83.4-93.0]) and plasma iron concentrations (mean [SD]: 0.82 μg/mL [0.33]) were in the normal physiological range (**Table 2**). Iron deficiency was generally low, with prevalence higher in females (7.8%) than in males (0.4%). The prevalence of iron deficiency in women of reproductive age (< 50 years) was 17.5%, whereas the prevalence in females in the age group 18–64 years was 14.8%. The risk of iron overload was also low. Out of 572 participants, 4 women of reproductive age had a ferritin concentration > 150 μg/L and 46 men or post-menopausal women had a ferritin concentration > 200 μg/L. Of these, 7 participants had a ferritin concentration > 250 μg/L. In the overall study population, the mean sTfR was 2.72 mg/L (SD 1.30), and the mean body iron stores were 11.7 mg/Kg (SD 3.7). Prevalence of inflammation was also low (2.3%).

**Table 2:** Iron status of participants of the Zurich SARS-CoV-2 vaccine cohort study overall and by sex and age group (n = 572).

Iron and inflammatory markers	Overall	Sex		Age group	
		Males	Females	18–64 years	65+ years
	n = 572	n = 252	n = 320	n = 307	n = 265
Plasma ferritin (μg/L)	88.1 (83.4-93.0)	115.3 (108.3-122.8)	71.2 (66.0-76.8)	75.9 (69.9-82.3)	104.7 (98.3-111.5)
Plasma iron (μg/mL)	0.82 (0.33)	0.82 (0.35)	0.82 (0.32)	0.84 (0.33)	0.80 (0.33)
Iron deficiency, n (%)	26 (4.5)	1 (0.4)	25 (7.8)	24 (7.8)	2 (0.8)
Soluble transferrin receptor (mg/L)	2.72 (1.30)	2.66 (1.41)	2.77 (1.20)	2.79 (1.28)	2.65 (1.31)
Body iron stores (mg/Kg)	11.7 (3.7)	13.0 (3.5)	10.7 (3.6)	11.0 (4.0)	12.5 (3.2)
Retinol binding protein (μmol/L)	1.03 (0.99-1.08)	1.06 (1.00-1.14)	1.01 (0.96-1.06)	1.00 (0.95-1.06)	1.07 (1.00-1.14)
CRP (mg/L)	0.44 (0.39-0.49)	0.43 (0.37-0.51)	0.44 (0.38-0.51)	0.33 (0.28-0.38)	0.61 (0.52-0.71)
AGP (g/L)	0.46 (0.18)	0.45 (0.19)	0.47 (0.17)	0.46 (0.17)	0.46 (0.20)
Inflammation, n (%)	13 (2.3)	7 (2.8)	6 (1.9)	0 (0.0)	13 (4.9)

Continuous variables are given as mean (SD) or geometric mean (95% CI), categorical variables as n (%).

Plasma iron values were based on a study sample of n = 562.

Iron deficiency was defined as plasma ferritin < 25 μg/L.

Inflammation was defined as CRP ≥ 5 mg/L or AGP ≥ 1 g/L.

AGP, alpha(1)-acid glycoprotein; CI, confidence interval; CRP, c-reactive protein; SD, standard deviation.

**Tables 3**, **4** show results of linear mixed-effect models exploring the longitudinal association of antibody and NAb levels with plasma ferritin and plasma iron, respectively, over 6 months. We found significant positive associations between plasma ferritin concentration at time of vaccination and antibody levels as well as NAb levels in both continuous and categorical analyses (**Table 3**). For every 50 μg/L increase in plasma ferritin concentration, we observed a 5.2% increase in Anti-S IgG antibodies, a 13.6% increase in Anti-Ancestral NAb, a 19.9% increase in Anti-Delta NAb, and a 16.7% increase in Anti-Omicron NAb over 6 months. Similarly, when exploring quartiles of plasma ferritin levels, individuals in the highest quartile (corresponding to a plasma ferritin level range of 141.0-250.0 μg/L) showed a 14.9% higher immune response for Anti-S IgG antibodies, and a 47.1%, 82.2%, and 75.1% higher immune response for Anti-Ancestral NAb, Anti-Delta NAb, and Anti-Omicron NAb, respectively, compared to those in the lowest quartile. We found no evidence for an association between plasma ferritin levels and Anti-S IgA antibodies.

**Table 3:** Longitudinal associations between plasma ferritin levels prior to vaccination and different immunity markers over 6 months.

Plasma ferritin	Anti-S IgA			Anti-S IgG			Anti-Ancestral NAb			Anti-Delta NAb			Anti-Omicron NAb		
	Exp (β)	β	95%CI	Exp (β)	β	95%CI	Exp (β)	β	95%CI	Exp (β)	β	95%CI	Exp (β)	β	95%CI
Continuous analysis															
Plasma ferritin	1.023	0.010	-0.022;0.042	1.052	0.022	0.009;0.035	1.136	0.055	0.006;0.105	1.199	0.079	0.028;0.702	1.167	0.067	0.017;0.118
Analysis by quartiles															
Q1 (4.3-62.1 μg/L)	1	0		1	0		1	0		1	0		1	0	
Q2 (62.2-96.3 μg/L)	1.035	0.015	-0.081;0.111	0.999	0.000	-0.040;0.040	0.909	-0.041	-0.176;0.094	1.017	0.007	-0.130;0.146	1.020	0.009	-0.128;0.146
Q3 (96.4-140.9 μg/L)	0.980	-0.009	-0.105;0.088	1.036	0.015	-0.025;0.055	0.967	-0.015	-0.154;0.126	1.083	0.035	-0.108;0.178	1.000	0.000	-0.141;0.142
Q4 (141.0-250.0 μg/L)	1.142	0.058	-0.047;0.163	1.149	0.060	0.017;0.104	1.471	0.168	0.001;0.334	1.822	0.261	0.090;0.431	1.751	0.243	0.074;0.411

Results were derived from linear mixed-effect models, using a random intercept for individuals and antibody or NAb levels as outcomes; models were adjusted for antibody level at baseline, vaccine type and number of vaccine doses received, time point of study visit, re-exposure at study visit, CRP, AGP, RBP, age, sex, smoking status. Complete results are presented in **Supplementary Tables S1** and **S2**.

Coefficients β are on the log10 scale; exp(β), i.e. 10^β^, represents the multiplicative factor changes in antibody or NAb levels associated with a 50 μg/L increase in plasma ferritin levels in continuous analyses or compared to plasma ferritin Q1 in categorical analyses.

Models were based on a study sample of n=563 for Anti-S IgA, n=563 for Anti-S IgG, n=212 for Anti-Ancestral NAb, n=212 for Anti-Delta NAb, n=212 for Anti-Omicron Nab.

AGP, alpha(1)-acid glycoprotein; CI, confidence intervals; CRP, c-reactive protein; NAb, neutralizing antibodies; Q, quartile; RBP, retinol binding protein.

**Table 4:** Longitudinal associations between plasma iron levels prior to vaccination and different immunity markers over 6 months.

Plasma iron	Anti-S IgA			Anti-S IgG			Anti-Ancestral NAb			Anti-Delta NAb			Anti-Omicron NAb		
	Exp (β)	β	95%CI	Exp (β)	β	95%CI	Exp (β)	β	95%CI	Exp (β)	β	95%CI	Exp (β)	β	95%CI
Continuous analysis															
Plasma iron	0.859	-0.066	-0.201;0.070	0.964	-0.016	-0.073;0.041	0.631	-0.200	-0.398;0.000	0.735	-0.134	-0.341;0.074	0.642	-0.192	-0.394;0.011
Analysis by quartiles															
Q1 (0.12-0.59 μg/mL)	1	0		1	0		1	0		1	0		1	0	
Q2 (0.59-0.81 μg/mL)	0.918	-0.037	-0.136;0.062	0.969	-0.014	-0.055;0.028	0.804	-0.095	-0.246;0.054	0.718	-0.144	-0.300;0.011	0.742	-0.130	-0.282;0.021
Q3 (0.81-1.06 μg/mL)	1.050	0.021	-0.093;0.136	0.984	-0.007	-0.055;0.041	0.672	-0.172	-0.351;0.005	0.666	-0.177	-0.361;0.008	0.616	-0.210	-0.389;-0.030
Q4 (1.06-1.91 μg/mL)	0.910	-0.041	-0.161;0.079	0.975	-0.011	-0.061;0.040	0.661	-0.180	-0.356;-0.003	0.663	-0.178	-0.361;0.005	0.625	-0.204	-0.382;-0.025

Results were derived from linear mixed-effect models, using a random intercept for individuals and antibody or NAb levels as outcomes; model were adjusted for antibody level at baseline, vaccine type and number of vaccine doses received, time point of study visit, time of the day of study visit, re-exposure at study visit, CRP, AGP, RBP, age, sex, smoking status. Complete results are presented in **Supplementary Tables S3** and **S4**.

Coefficients β are on the log10 scale; exp(β), i.e. 10^β^, represents the multiplicative factor changes in antibody or NAb levels associated with a 1 μg/mL increase in plasma iron levels in continuous analyses or compared to plasma iron Q1 in categorical analyses.

Models were based on a study sample of n=553 for Anti-S IgA, n=553 for Anti-S IgG, n=209 for Anti-Ancestral NAb, n=209 for Anti-Delta NAb, n=209 for Anti-Omicron NAb.

AGP, alpha(1)-acid glycoprotein; CI, confidence intervals; CRP, c-reactive protein; NAb, neutralizing antibodies; Q, quartile; RBP, retinol binding protein.

Analyses of plasma iron were generally less consistent across different markers of immunity (**Table 4**). No evidence for an association between plasma iron concentrations and antibody or NAb levels was observed when plasma iron was included as continuous variable in the models. When investigating quartiles of plasma iron levels, individuals in the highest quartile of plasma iron had a 33.9% lower antibody response for Anti-Ancestral NAb and a 37.5% lower antibody response for Anti-Omicron NAb levels compared to those in the lowest quartile. We found no evidence that quartiles of plasma iron levels were associated with Anti-S IgA antibodies, Anti-S IgG antibodies, and Anti-Delta NAb.

Results of covariates in all models showed a high level of consistency across iron parameters and markers of immunity (**Supplementary Tables S1-S4**). As expected, baseline antibody concentration was positively associated with the immune response. In addition, re-exposure to SARS-CoV-2, either by infection or subsequent vaccination, was associated with a markedly higher immune response. Receiving two doses of the Moderna mRNA-1273 vaccine was associated with a higher immune response, while receiving the Johnson & Johnson JNJ-78436735 vaccine was associated with a lower immune response compared to receiving two doses of either mRNA vaccine. Finally, the immune response following vaccination was decreased with increasing age and was lower in males compared to females.

We also explored the association of antibody and NAb levels with plasma ferritin and plasma iron at single time points of study follow-up. Results of models including plasma ferritin are shown in **Supplementary Table S5**. Consistent with the results of the longitudinal analyses, significant positive associations were observed for Anti-S IgG as well as for NAb against the Ancestral, Delta and Omicron viral variants, whereas no evidence for an association with plasma ferritin levels was observed for Anti-S IgA. For Anti-S IgG antibodies, we observed a 9.0% increase in antibody levels for every 50 μg/L increase in plasma ferritin concentration at 4 weeks, but no evidence for an association at 6 weeks, i.e., after participants received the second vaccine dose. Evidence for positive associations was again observed at 3 months and 6 months, where we observed a 3.8% and 5.3% increase in Anti-S IgG antibody levels for every 50 μg/L increase in plasma ferritin, respectively. In models investigating NAb, positive associations were observed at 3 months (19.0% increase for Anti-Ancestral, 28.6% for Anti-Delta and 28.7% for Anti-Omicron NAb for every 50 μg/L increase in plasma ferritin concentration). For plasma iron, no relevant trends were observed in analyses at individual time points of study follow-up (**Supplementary Table S6**). Generally, we found no evidence for associations between plasma iron concentrations and immunity markers, with a few exceptions.

In general, sensitivity analyses including comorbidity as an adjustment factor in the models, excluding participants with inflammation, not adjusting the models for CRP and AGP, and adjusting the analyses of NAb for seropositivity at baseline and knowledge of a prior infection instead of baseline Anti-S IgG level did not improve the model fit and did not meaningfully change the study results (**Supplementary Tables S7-S10**). Not adjusting the models for CRP and AGP and adjusting the NAb analyses for seropositivity at baseline and knowledge of a prior infection tended to move the plasma iron results slightly away from the null (**Supplementary Tables S9-S10**).

**Figure 1:**
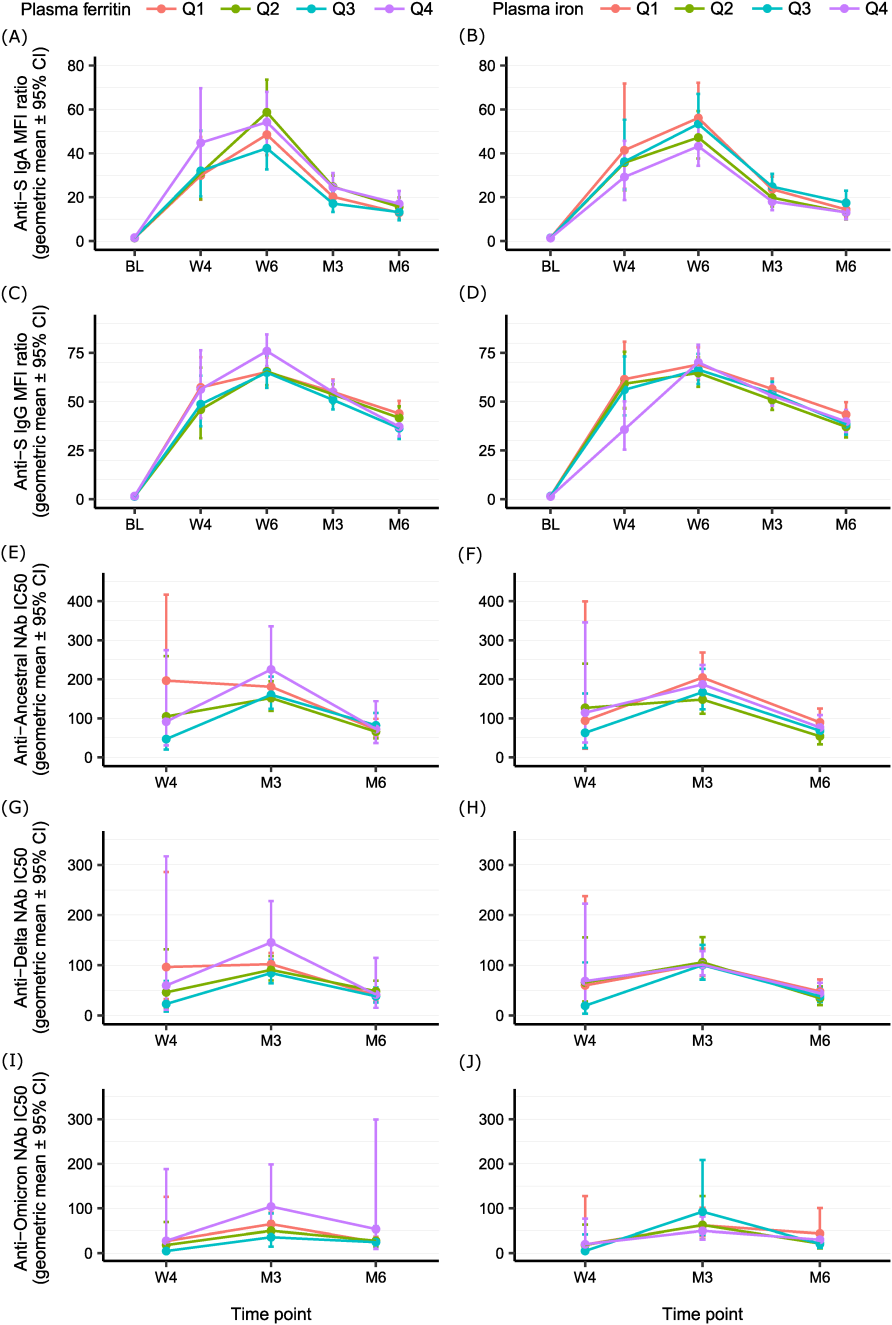
Temporal development of different immunity markers by plasma ferritin and plasma iron quartiles. **(A)** Anti-S IgA antibody levels by plasma ferritin quartiles (n=550). **(B)** Anti-S IgA antibody levels by plasma iron quartiles (n=540). **(C)** Anti-S IgG antibody levels by plasma ferritin quartiles (n=570). **(D)** Anti-S IgG antibody levels by plasma iron quartiles (n=560). **(E)** Anti-Ancestral NAb levels by plasma ferritin quartiles (n=184). **(F)** Anti-Ancestral NAb levels by plasma iron quartiles (n=181). **(G)** Anti-Delta NAb levels by plasma ferritin quartiles (n=138). **(H)** Anti-Delta NAb levels by plasma iron quartiles (n=136). **(I)** Anti-Omicron NAb levels by plasma ferritin quartiles (n=75). **(J)** Anti-Omicron NAb levels by plasma iron quartiles (n=73). Antibody level is expressed as geometric mean (95% CI) MFI ratio for Anti-S IgA and Anti-S IgG antibodies, and as geometric mean (95% CI) IC_50_ for Anti-Ancestral, Anti-Delta, and Anti-Omicron NAb. Time points in which participants reported a re-exposure or antibody levels increased (post-vaccination or potential undiagnosed SARS-CoV-2 infection) were excluded. CI, confidence interval; IC_50_, half maximal inhibitory concentration serum dilution; NAb, neutralizing antibodies; Q, quartiles.

Next, we estimated the half-life of the different markers of immune response based on linear decay models. The temporal development of different immunity markers by plasma ferritin and plasma iron quartiles is presented in **Figure 1**. Mean antibody levels at 6 months were high in the overall study population as well as across all plasma ferritin and plasma iron quartiles (**Figures 1A–D**, **Supplementary Table S11**). Mean NAb levels at 6 months were also high against the Ancestral, but lower against the Delta and Omicron viral variants (**Figures 1E–J**, **Supplementary Table S11**). It should be noted that the sample size for analyses of the Omicron viral variant was limited and the half-life of Anti-Omicron NAb could not be estimated.

The estimated half-lives in days by plasma ferritin levels are shown in **Table 5**. Generally, Anti-S IgG antibodies had a longer half-life (ranging from 110 to 150 days) than Anti-S IgA antibodies (ranging from 58 to 64 days) as well as Anti-Ancestral and Anti-Delta NAb (ranging from 55 to 85 days). For Anti-S IgG antibodies, the estimated decay time decreased with increasing plasma ferritin levels prior to vaccination. Compared to participants in the first (lowest) quartile of plasma ferritin levels (152.1 days, 95% CI 131.6,172.7), the half-life was shorter in participants in the third quartile (121.4 days, 95% CI 108.5,134.3, p-value=0.009) and fourth quartile (109.8 days, 95% CI 99.1,120.3, p-value<0.001) of plasma ferritin concentrations. This might seem counterintuitive, as a longer half-life is desirable and in previous analyses, higher plasma ferritin concentration was associated with higher Anti-S IgG antibody levels. In contrast, for Anti-Ancestral NAb, participants in the third quartile of plasma ferritin levels (81.6 days, 95% CI 61.7,101.5, p-value=0.002) showed a longer half-life compared to participants in the first quartile of plasma ferritin levels (54.6 days, 95% CI 47.4,61.8; **Table 5**).

**Table 5:** Decay estimation of different markers of immune response by plasma ferritin level prior to vaccination.

Immunity markers	Unadjusted half-life		Adjusted half-life	
	Days (95% CI)	P-value	Days (95% CI)	P-value
Anti-S IgA				
Plasma ferritin Q1 (4.3-62.1 μg/L)	63.9 (57.1;70.6)	Ref	62.8 (56.6;68.9)	Ref
Plasma ferritin Q2 (62.2-96.3 μg/L)	62.0 (55.3;68.7)	0.705	61.5 (55.4;67.6)	0.776
Plasma ferritin Q3 (96.4-140.9 μg/L)	57.9 (52.1;63.8)	0.194	58.4 (52.9;64.0)	0.301
Plasma ferritin Q4 (141.0-250.0 μg/L)	60.5 (53.9;67.0)	0.483	63.2 (56.6;69.8)	0.928
Anti-S IgG				
Plasma ferritin Q1 (4.3-62.1 μg/L)	148.8 (125.8;171.8)	Ref	152.1 (131.6;172.7)	Ref
Plasma ferritin Q2 (62.2-96.3 μg/L)	147.3 (127.0;167.6)	0.924	147.5 (129.9;165.1)	0.734
Plasma ferritin Q3 (96.4-140.9 μg/L)	117.2 (103.2;131.2)	0.015	121.4 (108.5;134.3)	0.009
Plasma ferritin Q4 (141.0-250.0 μg/L)	110.7 (98.4;122.9)	0.002	109.8 (99.4;120.3)	<0.001
Anti-Ancestral NAb				
Plasma ferritin Q1 (4.3-62.1 μg/L)	56.5 (47.2;65.8)	Ref	54.6 (47.4;61.8)	Ref
Plasma ferritin Q2 (62.2-96.3 μg/L)	69.8 (55.1;84.5)	0.118	66.6 (55.5;77.7)	0.061
Plasma ferritin Q3 (96.4-140.9 μg/L)	84.6 (58.6;110.7)	0.015	81.6 (61.7;101.5)	0.002
Plasma ferritin Q4 (141.0-250.0 μg/L)	70.1 (47.3;92.9)	0.222	69.3 (51.1;87.5)	0.089
Anti-Delta NAb				
Plasma ferritin Q1 (4.3-62.1 μg/L)	59.7 (48.2;71.3)	Ref	57.0 (48.2;65.7)	Ref
Plasma ferritin Q2 (62.2-96.3 μg/L)	73.2 (52.8;93.7)	0.229	66.5 (52.8;80.3)	0.226
Plasma ferritin Q3 (96.4-140.9 μg/L)	70.4 (49.1;91.6)	0.359	66.7 (51.2;82.2)	0.252
Plasma ferritin Q4 (141.0-250.0 μg/L)	63.4 (42.6;84.2)	0.756	60.9 (45.8;76.0)	0.647
Anti-Omicron NAb				
Plasma ferritin	Not estimable		Not estimable	

Results were derived from linear mixed-effect models, using a random intercept for individuals, antibody or NAb levels as outcomes, and including an interaction term between days since maximum antibody concentration and plasma ferritin quartiles.

Estimated half-life was calculated using the formula log(0.5)/β, where β is the model-derived estimate of the interaction term between days since maximum antibody concentration and plasma ferritin quartiles; 95% CI were calculated using the delta method.

Adjusted half-life was derived from models adjusted for maximum antibody concentration, vaccine type and number of vaccine doses received, CRP, AGP, RBP, age, sex, smoking status.

Models were based on a study sample of n=550 for Anti-S IgA, n=570 for Anti-S IgG, n=184 for Anti-Ancestral NAb, n=138 for Anti-Delta NAb.

AGP, alpha(1)-acid glycoprotein; CI, confidence intervals; CRP, c-reactive protein; NAb, neutralizing antibodies; Q: quartile; RBP, retinol binding protein.

Analogous results for plasma iron analyses are presented in **Table 6**. Again, Anti-S IgG antibodies had a longer half-life (ranging from 122 to 143 days) compared to Anti-S IgA antibodies (ranging from 60 to 65 days) as well as Anti-Ancestral and Anti-Delta NAb (ranging from 55 to 85 days). However, no clear trend was observed in analyses of plasma iron and generally, no differences were observed in the estimated half-life by plasma iron quartiles (**Table 6**).

**Table 6:** Decay estimation of different markers of immune response by plasma iron level prior to vaccination.

Immunity markers	Unadjusted half-life		Adjusted half-life	
	Days (95% CI)	p-value	Days (95% CI)	p-value
Anti-S IgA				
Plasma iron Q1 (0.12-0.59 μg/mL)	60.1 (53.8;66.5)	Ref	60.6 (54.7;66.6)	Ref
Plasma iron Q2 (0.59-0.81 μg/mL)	60.8 (54.2;67.4)	0.891	60.7 (54.6;66.8)	0.980
Plasma iron Q3 (0.81-1.06 μg/mL)	63.6 (56.6;70.6)	0.470	65.3 (58.5;72.2)	0.303
Plasma iron Q4 (1.06-1.91 μg/mL)	60.1 (53.7;66.5)	0.990	59.5 (53.7;65.4)	0.803
Anti-S IgG				
Plasma iron Q1 (0.12-0.59 μg/mL)	133.4 (116.0;150.7)	Ref	134.2 (119.1;149.3)	Ref
Plasma iron Q2 (0.59-0.81 μg/mL)	122.1 (107.2;137.0)	0.333	124.5 (111.1;137.9)	0.340
Plasma iron Q3 (0.81-1.06 μg/mL)	139.2 (118.8;159.6)	0.668	142.7 (124.2;161.3)	0.478
Plasma iron Q4 (1.06-1.91 μg/mL)	125.2 (109.2;141.2)	0.499	126.6 (112.4;140.7)	0.465
Anti-Ancestral NAb				
Plasma iron Q1 (0.12-0.59 μg/mL)	67.7 (51.9;83.4)	Ref	62.8 (51.7;74.0)	Ref
Plasma iron Q2 (0.59-0.81 μg/mL)	57.9 (46.2;69.6)	0.321	56.8 (47.6;65.9)	0.399
Plasma iron Q3 (0.81-1.06 μg/mL)	78.8 (56.9;100.6)	0.409	77.2 (60.0;94.5)	0.148
Plasma iron Q4 (1.06-1.91 μg/mL)	68.1 (52.9;83.4)	0.966	65.8 (53.9;77.6)	0.723
Anti-Delta NAb				
Plasma iron Q1 (0.12-0.59 μg/mL)	74.7 (55.0;94.3)	Ref	70.5 (56.0;85.0)	Ref
Plasma iron Q2 (0.59-0.81 μg/mL)	54.3 (42.2;66.4)	0.072	51.7 (42.8;60.5)	0.021
Plasma iron Q3 (0.81-1.06 μg/mL)	66.9 (48.0;85.8)	0.583	63.3 (49.3;77.2)	0.479
Plasma iron Q4 (1.06-1.91 μg/mL)	68.3 (51.3;85.3)	0.631	65.5 (52.7;78.2)	0.607
Anti-Omicron NAb				
Plasma iron	Not estimable		Not estimable	

Results were derived from linear mixed-effect models, using a random intercept for individuals, antibody or NAb levels as outcomes, and including an interaction term between days since maximum antibody concentration and plasma iron quartiles.

Estimated half-life was calculated using the formula log(0.5)/β, where β is the model-derived estimate of the interaction term between days since maximum antibody concentration and plasma iron quartiles; 95% CI were calculated using the delta method.

Adjusted half-life was derived from models adjusted for maximum antibody concentration, vaccine type and number of vaccine doses received, time of the day of study visit, CRP, AGP, RBP, age, sex, smoking status.

Models were based on a study sample of n=540 for Anti-S IgA, n=560 for Anti-S IgG, n=181 for Anti-Ancestral NAb, n=136 for Anti-Delta NAb.

AGP, alpha(1)-acid glycoprotein; CI, confidence intervals; CRP: c-reactive protein; NAb, neutralizing antibodies; Q, quartiles; RBP, retinol binding protein.

## 4 Discussion

In this iron-replete study population, plasma ferritin concentration at the time of vaccination was positively associated with Anti-S IgG antibodies, and Anti-Ancestral, Anti-Delta, and Anti-Omicron NAb over 6 months. Plasma iron results were less consistent and generally, we found no evidence for an association between plasma iron levels and immunity markers. Higher Anti-S IgG antibody levels from 6 weeks to 3 months resulted in shorter half-lives of these antibodies in participants in the third and fourth quartiles of plasma ferritin concentrations compared to those in the first quartile. Despite this, Anti-S IgG concentrations at 6 months remained high across all quartile groups.

Ferritin is a well-established positive acute-phase reactant. Therefore, we adjusted all our models for markers of inflammation. In addition, we conducted a sensitivity analysis including only participants without inflammation. In this sensitivity analysis, the associations between plasma ferritin and immunity markers were very similar to those observed in the main analyses (**Supplementary Table S8**). However, given the observational nature of this study, residual confounding cannot be entirely excluded, and our findings have to be interpreted with caution.

For every 50 μg/L increase in plasma ferritin concentration, we found a 5% increase in Anti-S IgG antibodies and an increase between 14-20% in NAb against the Ancestral, Delta and Omicron viral variants over 6 months. Similarly, individuals in the highest quartile of plasma ferritin showed a 15% increase in Anti-S IgG antibodies and an increase between 47-82% in NAb compared to individuals in the lowest quartile (**Table 3**). Despite the significant associations, differences in antibody responses by iron status were small in magnitude and minor compared to other established determinants of vaccination efficacy, such as vaccine type and baseline antibody levels (**Supplementary Tables S1-S4**). Mean antibody and NAb levels at 6 months were generally high irrespective of iron status (**Figure 1**, **Supplementary Table S11**). In addition, seropositivity (defined as MFI ratio > 6.5 for IgA and MFI ratio > 6.0 for IgG antibodies) and neutralizing capacity (defined as serum dilution IC_50_ >50 for Anti-Ancestral, Anti-Delta, and Anti-Omicron NAb) at 6 months were generally consistent across quartiles of iron status (**Supplementary Table S12**). This indicates that the clinical implications of our results may be limited.

As the prevalence of iron deficiency in this study was low (4.5% in the overall study population and 17.5% in women of reproductive age), our finding of a positive association of plasma ferritin with most of the considered markers of immunity is somewhat surprising. The analyses by plasma ferritin quartiles indicated higher Anti-S IgG antibody and NAb levels in the subgroup with highest plasma ferritin concentrations (141.0-250.0 µg/L) compared to participants with the lowest plasma ferritin concentrations (4.3-62.1 µg/L). This result could be due to a suboptimal antibody response in the reference group, showing significant differences only in comparison with the group most different from the reference, i.e. the highest quartile of the plasma ferritin distribution. A further possible explanation is a positive effect of high plasma ferritin in this population on the antibody response to vaccination. A recent comprehensive analysis of drivers of post-acute sequelae of SARS-CoV-2 (or “long COVID”) suggested that delayed resolution of inflammation-associated hypoferremia best discriminated patients reporting persistent symptoms (28). Thus, abnormal iron distribution and functional iron deficiency at time of infection appear to be important risk factors for an inappropriate immune response to SARS-CoV-2. These results are broadly consistent with our observation of an association between high plasma ferritin and high antibody responses to SARS-CoV-2 vaccination. In the IRONMAN prospective randomized trial, conducted at the height of the COVID-19 pandemic and including subjects with heart failure and iron deficiency (defined as transferrin saturation <20% or serum ferritin <100 µg/L), treatment with intravenous iron reduced the risk of hospitalization for infection (hazard ratio 0.76, 95% CI 0.49-0.98), and a secondary analysis suggested that the effect was particularly pronounced in patients with hypoferremia (29). This finding is consistent with the observation that iron overload in β-thalassemia patients infected with SARS-CoV-2 was protective against in-hospital complications and all-cause mortality compared to matched controls (30). In another study, high ferritin (>600 ng/mL) was found to be an independent predictor of antibody levels after administration of SARS-CoV-2 vaccines in hemodialysis patients (31). These studies support the concept that the absence of functional iron deficiency at the time of vaccination (irrespective of plasma ferritin levels) results in a stronger SARS-CoV-2 immune response (28) and call for further studies on the interaction between immunity and iron status in vulnerable population groups.

The hepcidin induced hypoferremia of inflammation has an innate short term protective role reducing the amount of systemic iron in circulation and limiting pathogen proliferation (32). While plasma iron was not associated with antibody response in the continuous analyses of our study, being in the highest quartile of plasma iron was associated with lower Anti-Ancestral NAb and Anti-Omicron NAb concentrations. A possible explanation for this effect is that subjects with higher plasma iron levels tended to have higher antibody levels prior to vaccination, resulting in a more blunted immune response to vaccines, an effect that has also been described for influenza vaccinations (27). This explanation is supported by a substantial shift away from the null in the association between plasma iron and NAb when the models were not adjusted for baseline antibody levels (**Supplementary Table S13**).

In the current study, the estimated decay time for Anti-S IgG antibodies decreased with increasing plasma ferritin levels. This appears to be in contrast with the effect detected in linear mixed-effect models investigating the longitudinal association between Anti-S IgG antibody levels and plasma ferritin concentrations, where we observed a significantly higher immune response in individuals with higher plasma ferritin levels. The shorter half-life of Anti-S IgG antibodies in the higher plasma ferritin quartiles likely resulted from higher maximum antibody concentrations in these individuals, leading to a more rapid decline in Anti-S IgG antibody concentrations over time. Despite this, mean Anti-S IgG concentration at 6 months was high, indicating a robust immune response (**Supplementary Table S11**). A somewhat opposite effect was observed for Anti-Ancestral NAb, where higher plasma ferritin levels tended to be associated with a longer NAb half-life. The reason for this discrepancy is not immediately clear, but it has been suggested that different B cell subtypes (long-lived plasma cells *vs* short-lived plasma blasts) may react differently to iron availability (33).

To our knowledge only a few studies have investigated the influence of iron status on the immune response following SARS-CoV-2 vaccination, with most studies only focusing on the short-term effectiveness of vaccination. Faizo et al. (14) observed no differences in antibody levels and neutralizing capacity when investigating the SARS-CoV-2 vaccine-induced immunity in iron-deficient *vs*. iron-replete controls. However, the assessment of iron status after rather than at the time of vaccination, the difference in time since vaccination, the limited sample size, and the sex imbalance between the study groups might have influenced the results of this study (14). Furthermore, in a randomized controlled trial providing ferric carboxymaltose to kidney transplant recipients with iron deficiency, no differences in humoral and cellular immune response following SARS-CoV-2 vaccination were observed between study groups after vaccination (34). When comparing iron-deficient and iron-replete individuals, Tene et al. (35) found a similar SARS-CoV-2 vaccine effectiveness and comparable hospitalization and mortality rates between study groups at 3 weeks after the second vaccination. Interestingly, although without evidence for statistically significant differences, this study also found that the one-dose vaccine effectiveness was numerically lower in individuals with iron deficiency compared to individuals without iron deficiency. Similar results were observed in an intervention study, where anemic Kenyan women who received intravenous iron before vaccination with the ChAdOx vaccine (Oxford-AstraZeneca COVID-19) showed higher IgG antibodies after the first vaccination, compared to those who did not received iron before vaccination, but no difference was observed after the second vaccination (unpublished data (33)). This is consistent with our study, as we found positive associations between plasma ferritin concentrations at time of vaccination and Anti-S IgG antibodies at week 4, i.e. after participants received the first vaccine dose, but not at week 6, i.e. after participants received the second vaccine dose. Nevertheless, evidence for positive associations was again observed at 3 months and 6 months in analyses including plasma ferritin as continuous variable.

In our study, we observed a clear antibody and NAb response shortly after SARS-CoV-2 vaccination irrespective of iron status (**Figure 1**). This is in line with previous studies reporting a high two-dose effectiveness of SARS-CoV-2 in both iron-replete and iron-deficient individuals (14, 35) as well as with previous efficacy trials (36, 37). These results may indicate that a timely use of SARS-CoV-2 vaccines in iron-deficient individuals might be more advantageous than delaying vaccination to correct iron deficiency, which contrasts the recommendation issued by the European Hematology Association. Although our study was not suited to investigate effects among iron-deficient individuals, the weaker immune response observed among individuals with low iron levels prior to vaccination with a SARS-CoV-2 mRNA vaccine, which is known to induce a strong antibody response, suggests that the impact of iron levels on the efficacy of other vaccines requires further investigation.

This study used data from a population-based representative cohort study, timely established after the beginning of the administration of SARS-CoV-2 vaccines in Switzerland. Analyses included data collected from the time of vaccination until 6 months post vaccination. We performed a comprehensive analysis considering different markers of immune response, and plasma ferritin and plasma iron as indicators of iron status. Iron parameters were measured in the baseline samples, which were collected immediately before receiving the first vaccine dose, reflecting iron status at time of vaccination. Additionally, Anti-S IgA and Anti-S IgG antibodies were also measured in the baseline samples, providing information on antibody levels prior to vaccination. In all analyses, results of covariates showed a high level of consistency across iron parameters and markers of immunity and were in agreement with predictors of SARS-CoV-2 immune response identified in previously published studies. However, this study also has some limitations. First, iron parameters were measured in plasma samples and concentrations of plasma iron could have been affected by hemoglobin iron potentially present in the samples as a consequence of hemolysis. However, we corrected plasma iron concentrations for hemoglobin iron by assessing the degree of hemolysis and comparing this to a calibration curve prepared from plasma and hemolyzed whole blood (22). In addition, since the timing of blood collection was not standardized in the Zurich SARS-CoV-2 Vaccine Cohort Study, variability in plasma iron concentration related to the circadian variation cannot be excluded. However, we adjusted all analyses of plasma iron for the time of the day of study visit. Second, only a few participants were iron-deficient, precluding the possibility to compare iron-deficient and iron-replete individuals in this study. Therefore, study findings cannot be reliably generalized to iron-deficient populations. Nevertheless, we divided participants into groups based on plasma ferritin and plasma iron quartiles, which enabled us to conduct analyses comparing individuals with a relatively low iron status, but with a sufficient number of participants. Third, as an acute phase protein, it has been speculated whether ferritin could be a trigger for further propagation of an inflammatory cascade rather than a sole marker of inflammation (38). While the former cannot be excluded in this study, our participants were in large part naive to SARS-CoV-2 before vaccination. Fourth, the measurement of antibody responses in this study relied on a Luminex-based assay and test accuracy might have influenced the assessment of antibody levels. However, an extensive validation of the Luminex assay was performed prior to the start of the study, resulting into high sensitivity and specificity and showing high levels of agreement with results of two commercially available assays (23). Fifth, neutralizing activity was quantified indirectly with a surrogate assay by measuring the competitive inhibition of the SARS-CoV-2 S protein binding to the Angiotensin Converting Enzyme 2 (ACE2) receptor. Nevertheless, validation of this assay resulted in very high sensitivity compared to assays of live viruses. In addition, this allowed the simultaneous assessment of Anti-Ancestral, Anti-Delta, and Anti-Omicron NAb (23). Sixth, NAb were measured only in a subset of study participants, leading to a relatively low sample size in these analyses, particularly for the Omicron viral variant in analyses of the decay time. Seventh, as antibody and NAb concentrations were measured at pre-defined time points of study follow-up, the full kinetic profile of the immune response was not available. Therefore, the participants’ true maximum antibody or NAb concentration may not have been captured by the study measures, which could have influenced the results of the decay analysis. Finally, cellular immune response to vaccination was not investigated in this study.

To conclude, in this predominantly iron-replete cohort with ferritin concentrations within the normal physiological range, higher plasma ferritin at the time of vaccination was associated with a more marked vaccination-induced immune response to SARS-CoV-2 over 6 months. Furthermore, shorter half-lives of Anti-S IgG antibodies were observed in participants in the third and fourth quartile of plasma ferritin concentration compared to participants in the lowest quartile. Despite this, mean Anti-S IgG concentration was higher from 6 weeks to 3 months and remained high at 6 months, indicating a robust immune response. Seropositivity and neutralizing capacity at 6 months were generally consistent across quartiles of iron status. In addition, differences in antibody concentrations due to iron status were minor compared with other well-known determinants of vaccination efficacy. Although the clinical implications of our findings appear to be limited, our results are consistent with the view that iron status is associated with a more marked immune response following SARS-CoV-2 vaccination. Further research, including prospective studies with relevant clinical endpoints and focusing on iron-deficient populations, is needed.

## Data Availability

The raw data supporting the conclusions of this article will be made available by the authors, without undue reservation.
