# Supplementary material for: Association of plasma ferritin and plasma iron at time of vaccination with the immune response to SARS-CoV-2 vaccination: a longitudinal cohort study
Source: Front Immunol. 2026 Mar 4;17:1764884. doi: 10.3389/fimmu.2026.1764884 (PMC12995634; doi:10.3389/fimmu.2026.1764884)
Supplement: Supplementary file 1 [file DataSheet1.pdf]

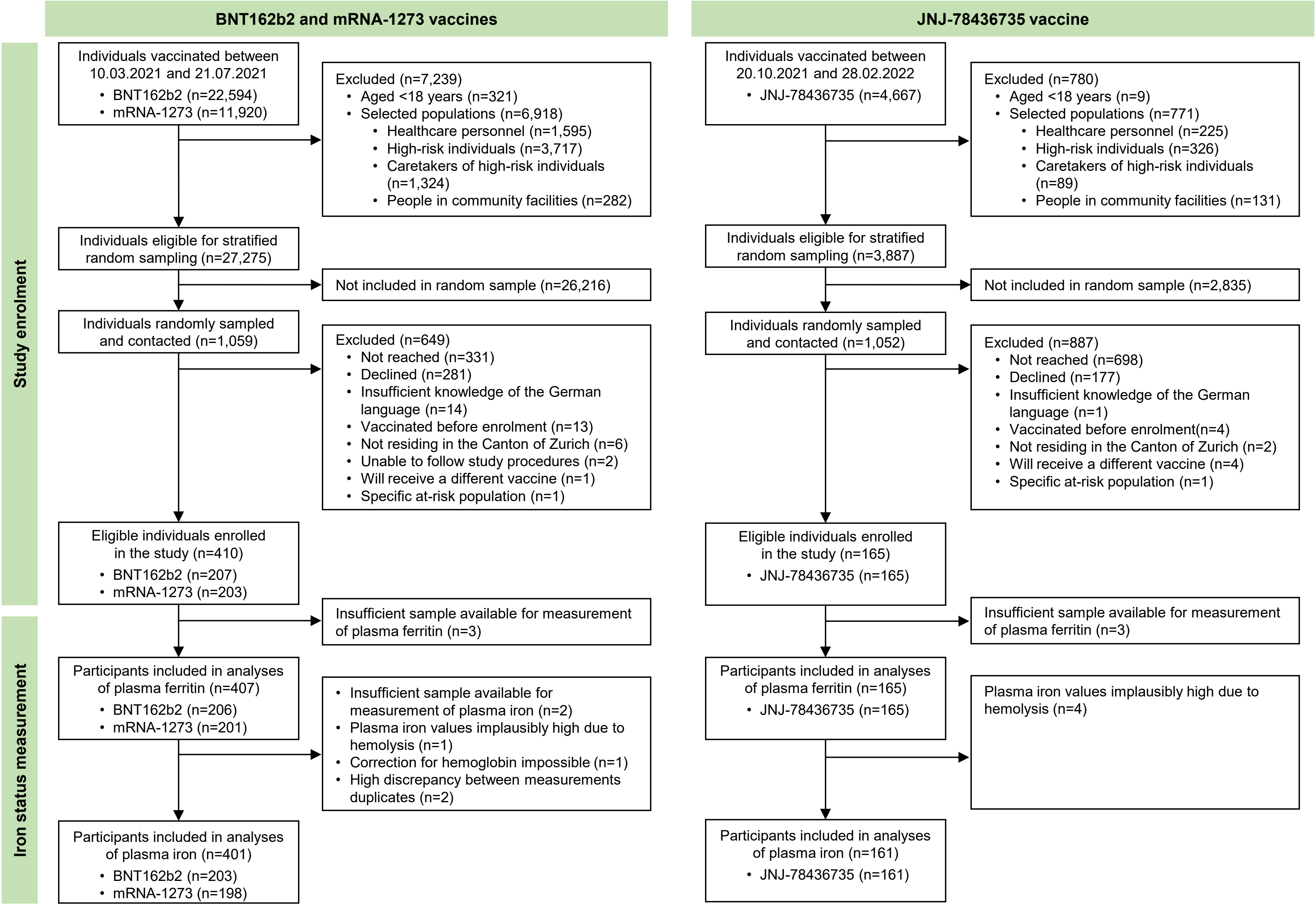

Figure S1: Participant flow diagram. Numbers for participants receiving the Pfizer-BioNTech BNT162b2 or the Moderna mRNA-1273 vaccines are shown on the left. Numbers for participants receiving the Johnson & Johnson JNJ-78436735 vaccine are shown on the right.

Table S1: Longitudinal associations between plasma ferritin levels prior to vaccination and different markers of immune response over 6 months (continuous)

	**Anti-S IgA**			**Anti-S IgG**			**Anti-Ancestral NAb**			**Anti-Delta NAb**			**Anti-Omicron NAb**		
	Exp (β)	β	95%CI	Exp (β)	β	95%CI	Exp (β)	β	95%CI	Exp (β)	β	95%CI	Exp (β)	β	95%CI
Plasma ferritin	1.023	0.010	-0.022 ; 0.042	1.052	0.022	0.009 ; 0.035	1.136	0.055	0.006 ; 0.105	1.199	0.079	0.028 ; 0.702	1.167	0.067	0.017 ; 0.118
Antibody levels at BL	3.400	0.532	0.444 ; 0.620	1.759	0.245	0.208 ; 0.282	4.437	0.647	0.526 ; 0.767	4.577	0.661	0.536 ; 0.783	5.501	0.740	0.616 ; 0.863
Vaccine type and dose															
BNT162b2 2 doses	1	0		1	0		1	0		1	0		1	0	
mRNA-1273 2 doses	1.900	0.279	0.201 ; 0.357	1.347	0.129	0.097 ; 0.162	1.805	0.256	0.139 ; 0.374	1.708	0.232	0.113 ; 0.352	1.424	0.153	0.035 ; 0.272
BNT162b2/mRNA-1273 1 dose	0.980	-0.009	-0.216 ; 0.198	0.771	-0.113	-0.201 ; -0.025	0.818	-0.087	-0.398 ; 0.224	0.920	-0.036	-0.352 ; 0.282	0.891	-0.050	-0.363 ; 0.265
JNJ-78436735 1 dose	0.379	-0.421	-0.504 ; -0.338	0.634	-0.198	-0.233 ; -0.163	0.762	-0.118	-0.242 ; 0.007	0.952	-0.022	-0.149 ; 0.107	0.807	-0.093	-0.221 ; 0.037
Re-exposure at visit	7.621	0.882	0.791 ; 0.972	2.757	0.440	0.389 ; 0.490	4.823	0.683	0.528 ; 0.832	4.480	0.651	0.484 ; 0.810	10.19	1.008	0.830 ; 1.173
CRP	1.028	0.012	-0.012 ; 0.036	0.985	-0.006	-0.016 ; 0.004	0.964	-0.016	-0.080 ; 0.049	0.992	-0.004	-0.070 ; 0.063	0.989	-0.005	-0.071 ; 0.061
AGP	1.140	0.057	-0.244 ; 0.357	1.217	0.085	-0.040 ; 0.210	0.961	-0.017	-0.515 ; 0.479	0.822	-0.085	-0.593 ; 0.422	0.798	-0.098	-0.604 ; 0.405
Retinol binding protein	0.903	-0.044	-0.140 ; 0.052	0.920	-0.036	-0.076 ; 0.004	0.981	-0.008	-0.170 ; 0.152	0.904	-0.044	-0.208 ; 0.121	0.952	-0.021	-0.184 ; 0.142
Age (per year increase)	1.000	0.000	-0.002 ; 0.002	0.996	-0.002	-0.003 ; -0.001	0.984	-0.007	-0.010 ; -0.004	0.982	-0.008	-0.011 ; -0.005	0.985	-0.006	-0.010 ; -0.003
Sex															
Female	1	0		1	0		1	0		1	0		1	0	
Male	1.085	0.035	-0.036 ; 0.106	0.832	-0.080	-0.109 ; -0.050	0.655	-0.184	-0.289 ; -0.078	0.612	-0.213	-0.321 ; -0.105	0.659	-0.181	-0.289 ; -0.074
Smoking status															
Non-smoker	1	0		1	0		1	0		1	0		1	0	
Ex-smoker	1.200	0.079	0.000 ; 0.158*	1.035	0.015	-0.018 ; 0.048	1.183	0.073	-0.049 ; 0.193	1.307	0.116	-0.008 ; 0.239	1.320	0.121	-0.003 ; 0.243
Smoker	0.945	-0.025	-0.113 ; 0.064	0.940	-0.027	-0.064 ; 0.010	0.781	-0.108	-0.229 ; 0.014	0.810	-0.092	-0.216 ; 0.033	0.904	-0.044	-0.168 ; 0.079

Results were derived from linear mixed-effect models, using a random intercept for individuals and antibody or NAb levels as outcomes; models were further adjusted for time point of study visit

Coefficients β are on the log10 scale; exp(β), i.e. 10^{β}, represents the multiplicative factor changes in antibody or NAb levels associated with a one unit increase in the independent variable or compared to the reference category. Plasma ferritin was rescaled to reflect changes in antibody or NAb levels associated with a 50 μg/L increase in plasma ferritin levels.

Models were based on a study sample of n=563 for Anti-S IgA, n=563 for Anti-S IgG, n=212 for Anti-Ancestral NAb, n=212 for Anti-Delta NAb, n=212 for Anti-Omicron NAb

For Anti-S IgA models, Anti-S IgA levels at baseline were used; for all other models, Anti-S IgG levels at baseline were used

*95%CI=0.0002;0.1583

AGP: alpha(1)-acid glycoprotein; BL: baseline; CI: confidence intervals; CRP: c-reactive protein; NAb: neutralizing antibodies

Table S2: Longitudinal associations between plasma ferritin levels prior to vaccination in quartiles and different markers of immune response over 6 months (categorical)

	Anti-S IgA			Anti-S IgG			Anti-Ancestral NAb			Anti-Delta NAb			Anti-Omicron NAb		
	Exp (β)	β	95%CI	Exp (β)	β	95%CI	Exp (β)	β	95%CI	Exp (β)	β	95%CI	Exp (β)	β	95%CI
Plasma ferritin															
Q1 (4.3-62.1 μg/L)	1	0		1	0		1	0		1	0		1	0	
Q2 (62.2-96.3 μg/L)	1.035	0.015	-0.081 ; 0.111	0.999	0.000	-0.040 ; 0.040	0.909	-0.041	-0.176 ; 0.094	1.017	0.007	-0.130 ; 0.146	1.020	0.009	-0.128 ; 0.146
Q3 (96.4-140.9 μg/L)	0.980	-0.009	-0.105 ; 0.088	1.036	0.015	-0.025 ; 0.055	0.967	-0.015	-0.154 ; 0.126	1.083	0.035	-0.108 ; 0.178	1.000	0.000	-0.141 ; 0.142
Q4 (141.0-250.0 μg/L)	1.142	0.058	-0.047 ; 0.163	1.149	0.060	0.017 ; 0.104	1.471	0.168	0.001 ; 0.334	1.822	0.261	0.090 ; 0.431	1.751	0.243	0.074 ; 0.411
Antibody levels at BL	3.396	0.531	0.443 ; 0.619	1.756	0.244	0.207 ; 0.281	4.329	0.636	0.515 ; 0.756	4.431	0.647	0.522 ; 0.769	5.305	0.725	0.601 ; 0.847
Vaccine type and dose															
BNT162b2 2 doses	1	0		1	0		1	0		1	0		1	0	
mRNA-1273 2 doses	1.906	0.280	0.202 ; 0.358	1.349	0.130	0.098 ; 0.163	1.809	0.258	0.140 ; 0.374	1.728	0.238	0.118 ; 0.357	1.439	0.158	0.041 ; 0.276
BNT162b2/mRNA-1273 1 dose	0.990	-0.005	-0.212 ; 0.203	0.778	-0.109	-0.197 ; -0.021	0.788	-0.103	-0.416 ; 0.211	0.929	-0.032	-0.351 ; 0.289	0.890	-0.050	-0.365 ; 0.266
JNJ-78436735 1 dose	0.380	-0.420	-0.503 ; -0.337	0.636	-0.197	-0.232 ; -0.162	0.769	-0.114	-0.237 ; 0.010	0.960	-0.018	-0.145 ; 0.111	0.814	-0.089	-0.216 ; 0.039
Re-exposure at visit	7.638	0.883	0.791 ; 0.973	2.765	0.442	0.390 ; 0.491	4.930	0.693	0.537 ; 0.841	4.580	0.661	0.493 ; 0.819	10.48	1.020	0.842 ; 1.184
CRP	1.026	0.011	-0.013 ; 0.035	0.985	-0.007	-0.017 ; 0.004	0.966	-0.015	-0.079 ; 0.049	0.991	-0.004	-0.070 ; 0.062	0.987	-0.006	-0.071 ; 0.060
AGP	1.161	0.065	-0.236 ; 0.366	1.193	0.077	-0.048 ; 0.202	0.806	-0.094	-0.595 ; 0.407	0.717	-0.145	-0.657 ; 0.367	0.707	-0.151	-0.658 ; 0.355
Retinol binding protein	0.896	-0.048	-0.143 ; 0.048	0.924	-0.034	-0.074 ; 0.006	1.035	0.015	-0.146 ; 0.175	0.951	-0.022	-0.186 ; 0.142	0.995	-0.002	-0.164 ; 0.160
Age (per year increase)	1.000	0.000	-0.002 ; 0.002	0.996	-0.002	-0.002 ; -0.001	0.985	-0.006	-0.010 ; -0.003	0.983	-0.008	-0.011 ; -0.004	0.986	-0.006	-0.009 ; -0.003
Sex															
Female	1	0		1	0		1	0		1	0		1	0	
Male	1.071	0.030	-0.041 ; 0.101	0.836	-0.078	-0.107 ; -0.048	0.653	-0.185	-0.291 ; -0.078	0.603	-0.220	-0.328 ; -0.110	0.644	-0.191	-0.299 ; -0.083
Smoking status															
Non-smoker	1	0		1	0		1	0		1	0		1	0	
Ex-smoker	1.202	0.080	0.001 ; 0.159	1.033	0.014	-0.019 ; 0.047	1.195	0.077	-0.044 ; 0.198	1.329	0.123	-0.001 ; 0.247	1.356	0.132	0.009 ; 0.254
Smoker	0.953	-0.021	-0.109 ; 0.068	0.943	-0.026	-0.063 ; 0.012	0.799	-0.098	-0.220 ; 0.024	0.831	-0.080	-0.205 ; 0.044	0.938	-0.028	-0.152 ; 0.095

Results were derived from linear mixed-effect models, using a random intercept for individuals and antibody or NAb levels as outcomes; models were further adjusted for time point of study visit

Coefficients β are on the log10 scale; exp(β), i.e. 10^{β}, represents the multiplicative factor changes in antibody or NAb levels associated with a one unit increase in the independent variable or compared to the reference category.

Models were based on a study sample of n=563 for Anti-S IgA, n=563 for Anti-S IgG, n=212 for Anti-Ancestral NAb, n=212 for Anti-Delta NAb, n=212 for Anti-Omicron NAb

For Anti-S IgA models, Anti-S IgA levels at baseline were used; for all other models, Anti-S IgG levels at baseline were used

AGP: alpha(1)-acid glycoprotein; BL: baseline; CI: confidence intervals; CRP: c-reactive protein; NAb: neutralizing antibodies; Q: quartiles

Table S3: Longitudinal associations between plasma iron levels prior to vaccination and different markers of immune response over 6 months (continuous)

	Anti-S IgA			Anti-S IgG			Anti-Ancestral NAb			Anti-Delta NAb			Anti-Omicron NAb		
	Exp (β)	β	95%CI	Exp (β)	β	95%CI	Exp (β)	β	95%CI	Exp (β)	β	95%CI	Exp (β)	β	95%CI
Plasma iron	0.859	-0.066	-0.201 ; 0.070	0.964	-0.016	-0.073 ; 0.041	0.631	-0.200	-0.398 ; 0.000*	0.735	-0.134	-0.341 ; 0.074	0.642	-0.192	-0.394 ; 0.011
Antibody levels at BL	3.386	0.530	0.441 ; 0.618	1.740	0.241	0.203 ; 0.278	4.150	0.618	0.491 ; 0.744	4.245	0.628	0.495 ; 0.759	4.879	0.688	0.558 ; 0.817
Vaccine type and dose															
BNT162b2 2 doses	1	0		1	0		1	0		1	0		1	0	
mRNA-1273 2 doses	1.896	0.278	0.200 ; 0.356	1.351	0.131	0.098 ; 0.163	1.938	0.287	0.169 ; 0.405	1.842	0.265	0.142 ; 0.388	1.542	0.188	0.069 ; 0.307
BNT162b2/mRNA-1273 1 dose	0.988	-0.005	-0.212 ; 0.201	0.779	-0.108	-0.197 ; -0.020	0.948	-0.023	-0.340 ; 0.295	1.067	0.028	-0.302 ; 0.359	1.093	0.039	-0.281 ; 0.360
JNJ-78436735 1 dose	0.377	-0.423	-0.507 ; -0.340	0.632	-0.199	-0.235 ; -0.164	0.797	-0.099	-0.224 ; 0.028	1.018	0.008	-0.124 ; 0.141	0.860	-0.065	-0.195 ; 0.066
Re-exposure at visit	7.452	0.872	0.780 ; 0.963	2.753	0.440	0.388 ; 0.490	4.984	0.698	0.541 ; 0.848	4.603	0.663	0.494 ; 0.824	10.46	1.019	0.839 ; 1.186
CRP	1.025	0.011	-0.014 ; 0.036	0.989	-0.005	-0.015 ; 0.006	0.954	-0.020	-0.086 ; 0.046	0.988	-0.005	-0.074 ; 0.064	0.971	-0.013	-0.080 ; 0.055
AGP	1.197	0.078	-0.234 ; 0.390	1.186	0.074	-0.057 ; 0.204	1.132	0.054	-0.470 ; 0.577	0.865	-0.063	-0.610 ; 0.482	1.035	0.015	-0.519 ; 0.545
Retinol binding protein	0.933	-0.030	-0.134 ; 0.074	0.946	-0.024	-0.068 ; 0.019	1.182	0.072	-0.095 ; 0.239	1.076	0.032	-0.142 ; 0.205	1.132	0.054	-0.115 ; 0.222
Age (per year increase)	1.000	0.000	-0.002 ; 0.002	0.996	-0.002	-0.002 ; -0.001	0.984	-0.007	-0.010 ; -0.004	0.984	-0.007	-0.010 ; -0.004	0.987	-0.006	-0.009 ; -0.003
Sex															
Female	1	0		1	0		1	0		1	0		1	0	
Male	1.102	0.042	-0.025 ; 0.109	0.863	-0.064	-0.092 ; -0.036	0.694	-0.159	-0.259 ; -0.058	0.685	-0.164	-0.269 ; -0.059	0.721	-0.142	-0.244 ; -0.039
Smoking status															
Non-smoker	1	0		1	0		1	0		1	0		1	0	
Ex-smoker	1.184	0.073	-0.007 ; 0.153	1.038	0.016	-0.017 ; 0.050	1.199	0.079	-0.044 ; 0.200	1.273	0.105	-0.023 ; 0.232	1.315	0.119	-0.006 ; 0.242
Smoker	0.942	-0.026	-0.115 ; 0.063	0.941	-0.027	-0.064 ; 0.011	0.779	-0.108	-0.231 ; 0.014	0.799	-0.097	-0.225 ; 0.030	0.905	-0.043	-0.168 ; 0.080

Results were derived from linear mixed-effect models, using a random intercept for individuals and antibody or NAb levels as outcomes; models were further adjusted for time point of study visit and time of the day of study visit

Coefficients β are on the log10 scale; exp(β), i.e. 10^{β}, represents the multiplicative factor changes in antibody or NAb levels associated with a one unit increase in the independent variable or compared to the reference category.

Models were based on a study sample of n=553 for Anti-S IgA, n=553 for Anti-S IgG, n=209 for Anti-Ancestral NAb, n=209 for Anti-Delta NAb, n=209 for Anti-Omicron NAb

For Anti-S IgA models, Anti-S IgA levels at baseline were used; for all other models, Anti-S IgG levels at baseline were used

*95%CI=-0.3984;-0.0004

AGP: alpha(1)-acid glycoprotein; BL: baseline; CI: confidence intervals; CRP: c-reactive protein; NAb: neutralizing antibodies

Table S4: Longitudinal associations between plasma iron levels prior to vaccination in quartiles and different markers of immune response over 6 months (categorical)

	Anti-S IgA			Anti-S IgG			Anti-Ancestral NAb			Anti-Delta NAb			Anti-Omicron NAb		
	Exp (β)	β	95%CI	Exp (β)	β	95%CI	Exp (β)	β	95%CI	Exp (β)	β	95%CI	Exp (β)	β	95%CI
Plasma iron															
Q1 (0.12-0.59 μg/mL)	1	0		1	0		1	0		1	0		1	0	
Q2 (0.59-0.81 μg/mL)	0.918	-0.037	-0.136 ; 0.062	0.969	-0.014	-0.055 ; 0.028	0.804	-0.095	-0.246 ; 0.054	0.718	-0.144	-0.300 ; 0.011	0.742	-0.130	-0.282 ; 0.021
Q3 (0.81-1.06 μg/mL)	1.050	0.021	-0.093 ; 0.136	0.984	-0.007	-0.055 ; 0.041	0.672	-0.172	-0.351 ; 0.005	0.666	-0.177	-0.361 ; 0.008	0.616	-0.210	-0.389 ; -0.030
Q4 (1.06-1.91 μg/mL)	0.910	-0.041	-0.161 ; 0.079	0.975	-0.011	-0.061 ; 0.040	0.661	-0.180	-0.356 ; -0.003	0.663	-0.178	-0.361 ; 0.005	0.625	-0.204	-0.382 ; -0.025
Antibody levels at BL	3.386	0.530	0.441 ; 0.618	1.741	0.241	0.203 ; 0.279	4.206	0.624	0.498 ; 0.748	4.241	0.627	0.497 ; 0.756	4.911	0.691	0.562 ; 0.818
Vaccine type and dose															
BNT162b2 2 doses	1	0		1	0		1	0		1	0		1	0	
mRNA-1273 2 doses	1.885	0.275	0.197 ; 0.354	1.349	0.130	0.097 ; 0.163	1.888	0.276	0.158 ; 0.393	1.785	0.252	0.130 ; 0.373	1.495	0.175	0.056 ; 0.293
BNT162b2/mRNA-1273 1 dose	1.013	0.006	-0.202 ; 0.213	0.778	-0.109	-0.198 ; -0.020	0.905	-0.043	-0.360 ; 0.275	1.057	0.024	-0.303 ; 0.353	1.051	0.022	-0.296 ; 0.341
JNJ-78436735 1 dose	0.375	-0.426	-0.509 ; -0.342	0.632	-0.200	-0.235 ; -0.164	0.788	-0.103	-0.228 ; 0.023	1.019	0.008	-0.122 ; 0.140	0.856	-0.068	-0.196 ; 0.063
Re-exposure at visit	7.448	0.872	0.780 ; 0.962	2.755	0.440	0.388 ; 0.490	4.934	0.693	0.536 ; 0.843	4.603	0.663	0.494 ; 0.823	10.37	1.016	0.834 ; 1.181
CRP	1.027	0.012	-0.013 ; 0.036	0.989	-0.005	-0.015 ; 0.006	0.964	-0.016	-0.082 ; 0.050	0.992	-0.004	-0.072 ; 0.065	0.981	-0.008	-0.075 ; 0.059
AGP	1.118	0.048	-0.266 ; 0.362	1.183	0.073	-0.059 ; 0.204	1.091	0.038	-0.480 ; 0.555	0.933	-0.030	-0.567 ; 0.505	1.051	0.022	-0.504 ; 0.543
Retinol binding protein	0.901	-0.045	-0.148 ; 0.058	0.940	-0.027	-0.070 ; 0.016	1.216	0.085	-0.085 ; 0.254	1.138	0.056	-0.120 ; 0.232	1.198	0.079	-0.093 ; 0.249
Age (per year increase)	1.000	0.000	-0.002 ; 0.002	0.996	-0.002	-0.002 ; -0.001	0.985	-0.007	-0.010 ; -0.004	0.984	-0.007	-0.010 ; -0.004	0.987	-0.006	-0.009 ; -0.003
Sex															
Female	1	0		1	0		1	0		1	0		1	0	
Male	1.100	0.042	-0.025 ; 0.108	0.862	-0.064	-0.092 ; -0.036	0.696	-0.157	-0.258 ; -0.056	0.679	-0.168	-0.273 ; -0.063	0.721	-0.142	-0.244 ; -0.040
Smoking status															
Non-smoker	1	0		1	0		1	0		1	0		1	0	
Ex-smoker	1.183	0.073	-0.007 ; 0.153	1.040	0.017	-0.017 ; 0.051	1.196	0.078	-0.045 ; 0.200	1.298	0.113	-0.014 ; 0.240	1.324	0.122	-0.003 ; 0.245
Smoker	0.952	-0.021	-0.110 ; 0.067	0.942	-0.026	-0.063 ; 0.012	0.773	-0.112	-0.235 ; 0.011	0.800	-0.097	-0.224 ; 0.030	0.900	-0.046	-0.170 ; 0.078

Results were derived from linear mixed-effect models, using a random intercept for individuals and antibody or NAb levels as outcomes; models were further adjusted for time point of study visit and time of the day of study visit

Coefficients β are on the log10 scale; exp(β), i.e. 10^{β}, represents the multiplicative factor changes in antibody or NAb levels associated with a one unit increase in the independent variable or compared to the reference category.

Models were based on a study sample of n=553 for Anti-S IgA, n=553 for Anti-S IgG, n=209 for Anti-Ancestral NAb, n=209 for Anti-Delta NAb, n=209 for Anti-Omicron NAb

For Anti-S IgA models, Anti-S IgA levels at baseline were used; for all other models, Anti-S IgG levels at baseline were used

AGP: alpha(1)-acid glycoprotein; BL: baseline; CI: confidence intervals; CRP: c-reactive protein; NAb: neutralizing antibodies; Q: quartiles

Table S5: Associations between plasma ferritin levels prior to vaccination and different markers of immune response at single time points of study follow-up

	**Anti-S IgA**			**Anti-S IgG**			**Anti-Ancestral NAb**			**Anti-Delta NAb**			**Anti-Omicron NAb**		
	Exp (β)	β	95%CI	Exp (β)	β	95%CI	Exp (β)	β	95%CI	Exp (β)	β	95%CI	Exp (β)	β	95%CI
**4 weeks**															
Plasma ferritin	1.048	0.021	-0.019 ; 0.060	1.090	0.038	0.016 ; 0.059	1.085	0.035	-0.105 ; 0.323	1.124	0.051	-0.023 ; 0.125	1.110	0.045	-0.024 ; 0.114
Plasma ferritin quartiles															
Q1 (4.3-62.1 μg/L)	1	0		1	0		1	0		1	0		1	0	
Q2 (62.2-96.3 μg/L)	1.012	0.005	-0.115 ; 0.125	1.035	0.015	-0.051 ; 0.081	0.841	-0.075	-0.266 ; 0.116	1.022	0.009	-0.193 ; 0.212	1.138	0.056	-0.133 ; 0.245
Q3 (96.4-140.9 μg/L)	1.052	0.022	-0.100 ; 0.144	1.138	0.056	-0.011 ; 0.123	0.859	-0.066	-0.263 ; 0.131	1.043	0.018	-0.190 ; 0.227	1.042	0.018	-0.177 ; 0.213
Q4 (141.0-250.0 μg/L)	1.208	0.082	-0.049 ; 0.213	1.273	0.105	0.033 ; 0.177	1.385	0.141	-0.093 ; 0.376	1.609	0.207	-0.041 ; 0.454	1.746	0.242	0.011 ; 0.473
**6 weeks**															
Plasma ferritin	0.964	-0.016	-0.052 ; 0.020	1.031	0.013	-0.003 ; 0.030									
Plasma ferritin quartiles															
Q1 (4.3-62.1 μg/L)	1	0		1	0										
Q2 (62.2-96.3 μg/L)	0.954	-0.021	-0.129 ; 0.087	0.958	-0.019	-0.068 ; 0.030									
Q3 (96.4-140.9 μg/L)	0.865	-0.063	-0.172 ; 0.046	1.000	0.000	-0.049 ; 0.050									
Q4 (141.0-250.0 μg/L)	0.920	-0.036	-0.154 ; 0.082	1.086	0.036	-0.018 ; 0.089									
**3 months**															
Plasma ferritin	1.043	0.018	-0.021 ; 0.057	1.038	0.016	0.000 ; 0.032*	1.190	0.075	0.016 ; 0.135	1.286	0.109	0.050 ; 0.168	1.287	0.109	0.038 ; 0.181
Plasma ferritin quartiles															
Q1 (4.3-62.1 μg/L)	1	0		1	0		1	0		1	0		1	0	
Q2 (62.2-96.3 μg/L)	1.125	0.051	-0.066 ; 0.168	1.024	0.010	-0.037 ; 0.058	0.888	-0.052	-0.213 ; 0.110	0.914	-0.039	-0.199 ; 0.121	0.881	-0.055	-0.250 ; 0.140
Q3 (96.4-140.9 μg/L)	0.902	-0.045	-0.163 ; 0.073	0.981	-0.008	-0.056 ; 0.039	1.028	0.012	-0.155 ; 0.179	1.161	0.065	-0.101 ; 0.230	1.106	0.044	-0.159 ; 0.247
Q4 (141.0-250.0 μg/L)	1.295	0.112	-0.016 ; 0.241	1.123	0.051	-0.001 ; 0.102	1.631	0.212	0.014 ; 0.411	2.143	0.331	0.134 ; 0.528	2.069	0.316	0.076 ; 0.556
**6 months**															
Plasma ferritin	1.043	0.018	-0.026 ; 0.063	1.053	0.022	0.003 ; 0.042	1.112	0.046	-0.027 ; 0.119	1.164	0.066	-0.006 ; 0.138	1.060	0.025	-0.044 ; 0.094
Plasma ferritin quartiles															
Q1 (4.3-62.1 μg/L)	1	0		1	0		1	0		1	0		1	0	
Q2 (62.2-96.3 μg/L)	1.146	0.059	-0.074 ; 0.193	1.007	0.003	-0.056 ; 0.062	1.169	0.068	-0.139 ; 0.275	1.302	0.115	-0.089 ; 0.318	1.218	0.086	-0.110 ; 0.281
Q3 (96.4-140.9 μg/L)	1.176	0.071	-0.064 ; 0.205	1.073	0.031	-0.029 ; 0.090	1.380	0.140	-0.080 ; 0.360	1.465	0.166	-0.050 ; 0.382	1.204	0.081	-0.126 ; 0.288
Q4 (141.0-250.0 μg/L)	1.263	0.102	-0.045 ; 0.248	1.151	0.061	-0.004 ; 0.126	1.200	0.079	-0.176 ; 0.335	1.477	0.169	-0.082 ; 0.421	1.129	0.053	-0.188 ; 0.294

Results were derived from linear regression models, using antibody or NAb levels as outcomes; models were adjusted for antibody levels at BL, vaccine type and number of vaccine doses received, re-exposure at corresponding time point of study visit, CRP, AGP, RBP, age, sex, smoking status

Coefficients β are on the log10 scale; exp(β), i.e. 10^{β}, represents the multiplicative factor changes in antibody or NAb levels associated with a 50 μg/L increase in plasma ferritin levels in continuous analyses or compared to plasma ferritin Q1 in categorical analyses

4 weeks: models were based on a study sample of n=556 for Anti-S IgA, n=556 for Anti-S IgG, n=208 for Anti-Ancestral NAb, n=208 for Anti-Delta NAb, n=208 for Anti-Omicron NAb

6 weeks: models were based on a study sample of n=540 for Anti-S IgA, n=540 for Anti-S IgG; no measurements of NAb were performed at 6 weeks

3 months: models were based on a study sample of n=546 for Anti-S IgA, n=546 for Anti-S IgG, n=210 for Anti-Ancestral NAb, n=210 for Anti-Delta NAb, n=209 for Anti-Omicron NAb

6 months: models were based on a study sample of n=544 for Anti-S IgA, n=544 for Anti-S IgG, n=139 for Anti-Ancestral NAb, n=139 for Anti-Delta NAb, n=139 for Anti-Omicron NAb

For Anti-S IgA models, Anti-S IgA levels at baseline were used; for all other models, Anti-S IgG levels at baseline were used

*95%CI=0.0003;0.0317

AGP: alpha(1)-acid glycoprotein; BL: baseline; CI: confidence intervals; CRP: c-reactive protein; NAb: neutralizing antibodies; Q: quartiles; RBP: retinol binding protein

Table S6: Associations between plasma iron levels prior to vaccination and different markers of immune response at single time points of study follow-up

	Anti-S IgA			Anti-S IgG			Anti-Ancestral NAb			Anti-Delta NAb			Anti-Omicron NAb		
	Exp (β)	β	95%CI	Exp (β)	β	95%CI	Exp (β)	β	95%CI	Exp (β)	β	95%CI	Exp (β)	β	95%CI
**4 weeks**															
Plasma iron	0.855	-0.068	-0.236 ; 0.100	0.865	-0.063	-0.156 ; 0.029	0.474	-0.324	-0.599 ; -0.049	0.616	-0.210	-0.504 ; 0.083	0.572	-0.243	-0.517 ; 0.032
Plasma iron quartiles															
Q1 (0.12-0.59 μg/mL)	1	0		1	0		1	0		1	0		1	0	
Q2 (0.59-0.81 μg/mL)	1.041	0.017	-0.107 ; 0.141	1.006	0.003	-0.066 ; 0.071	0.956	-0.019	-0.231 ; 0.192	0.907	-0.043	-0.267 ; 0.182	0.968	-0.014	-0.223 ; 0.195
Q3 (0.81-1.06 μg/mL)	1.183	0.073	-0.069 ; 0.215	1.006	0.003	-0.076 ; 0.081	0.700	-0.155	-0.404 ; 0.095	0.738	-0.132	-0.397 ; 0.133	0.722	-0.141	-0.388 ; 0.106
Q4 (1.06-1.91 μg/mL)	1.026	0.011	-0.138 ; 0.160	0.961	-0.017	-0.099 ; 0.065	0.613	-0.212	-0.460 ; 0.035	0.623	-0.206	-0.469 ; 0.058	0.592	-0.228	-0.473 ; 0.017
**6 weeks**															
Plasma iron	0.811	-0.091	-0.244 ; 0.062	1.072	0.030	-0.040 ; 0.100									
Plasma iron quartiles															
Q1 (0.12-0.59 μg/mL)	1	0		1	0										
Q2 (0.59-0.81 μg/mL)	0.970	-0.013	-0.127 ; 0.100	1.022	0.009	-0.042 ; 0.061									
Q3 (0.81-1.06 μg/mL)	0.975	-0.011	-0.140 ; 0.118	0.991	-0.004	-0.063 ; 0.055									
Q4 (1.06-1.91 μg/mL)	0.886	-0.053	-0.190 ; 0.084	1.040	0.017	-0.045 ; 0.079									
**3 months**															
Plasma iron	0.949	-0.023	-0.189 ; 0.144	1.025	0.011	-0.056 ; 0.078	0.662	-0.179	-0.419 ; 0.061	0.744	-0.128	-0.373 ; 0.116	0.669	-0.175	-0.471 ; 0.122
Plasma iron quartiles															
Q1 (0.12-0.59 μg/mL)	1	0		1	0		1	0		1	0		1	0	
Q2 (0.59-0.81 μg/mL)	0.927	-0.033	-0.155 ; 0.089	0.984	-0.007	-0.056 ; 0.042	0.782	-0.107	-0.287 ; 0.074	0.750	-0.125	-0.309 ; 0.059	0.820	-0.086	-0.307 ; 0.134
Q3 (0.81-1.06 μg/mL)	1.134	0.055	-0.086 ; 0.195	0.995	-0.002	-0.059 ; 0.054	0.651	-0.187	-0.401 ; 0.028	0.655	-0.184	-0.402 ; 0.035	0.568	-0.245	-0.507 ; 0.016
Q4 (1.06-1.91 μg/mL)	0.917	-0.037	-0.185; 0.110	0.985	-0.006	-0.066 ; 0.053	0.665	-0.177	-0.392 ; 0.038	0.700	-0.155	-0.374 ; 0.064	0.697	-0.157	-0.423 ; 0.109
**6 months**															
Plasma iron	0.840	-0.076	-0.263 ; 0.112	0.978	-0.010	-0.093 ; 0.074	1.111	0.046	-0.259 ; 0.350	1.153	0.062	-0.238 ; 0.362	0.951	-0.022	-0.308 ; 0.264
Plasma iron quartiles															
Q1 (0.12-0.59 μg/mL)	1	0		1	0		1	0		1	0		1	0	
Q2 (0.59-0.81 μg/mL)	0.799	-0.098	-0.236 ; 0.041	0.911	-0.041	-0.102 ; 0.021	0.520	-0.284	-0.513 ; -0.054	0.426	-0.371	-0.592 ; -0.149	0.435	-0.361	-0.573 ; -0.149
Q3 (0.81-1.06 μg/mL)	0.974	-0.011	-0.170 ; 0.147	0.983	-0.008	-0.078 ; 0.063	0.593	-0.227	-0.493 ; 0.039	0.569	-0.245	-0.501 ; 0.011	0.594	-0.226	-0.471 ; 0.019
Q4 (1.06-1.91 μg/mL)	0.823	-0.085	-0.251 ; 0.081	0.969	-0.014	-0.088 ; 0.060	0.758	-0.120	-0.380 ; 0.139	0.738	-0.132	-0.382 ; 0.118	0.681	-0.167	-0.406 ; 0.072

Results were derived from linear regression models, using antibody or NAb levels as outcomes; models were adjusted for antibody levels at BL, vaccine type and number of vaccine doses received, re-exposure at corresponding time point of study visit, time of the day of study visit, CRP, AGP, RBP, age, sex, smoking status

Coefficients β are on the log10 scale; exp(β), i.e. 10^{β}, represents the multiplicative factor changes in antibody or NAb levels associated with a 1 μg/mL increase in plasma iron levels in continuous analyses or compared to plasma iron Q1 in categorical analyses

4 weeks: models were based on a study sample of n=547 for Anti-S IgA, n=547 for Anti-S IgG, n=205 for Anti-Ancestral NAb, n=205 for Anti-Delta NAb, n=205 for Anti-Omicron NAb

6 weeks: models were based on a study sample of n=531 for Anti-S IgA, n=531 for Anti-S IgG; no measurements of NAb were performed at 6 weeks

3 months: models were based on a study sample of n=538 for Anti-S IgA, n=538 for Anti-S IgG, n=207 for Anti-Ancestral NAb, n=207 for Anti-Delta NAb, n=206 for Anti-Omicron NAb
6 months: models were based on a study sample of n=535 for Anti-S IgA, n=535 for Anti-S IgG, n=139 for Anti-Ancestral NAb, n=139 for Anti-Delta NAb, n=139 for Anti-Omicron NAb
For Anti-S IgA models, Anti-S IgA levels at baseline were used; for all other models, Anti-S IgG levels at baseline were used
AGP: alpha(1)-acid glycoprotein; BL: baseline; CI: confidence intervals; CRP: c-reactive protein; NAb: neutralizing antibodies; Q: quartiles; RBP: retinol binding protein

Table S7: Longitudinal associations between plasma ferritin or plasma iron levels prior to vaccination and different immunity markers over 6 months, adjusting linear mixed-effects models for comorbidities

	Anti-S IgA			Anti-S IgG			Anti-Ancestral NAb			Anti-Delta NAb			Anti-Omicron NAb		
	Exp (β)	β	95%CI	Exp (β)	β	95%CI	Exp (β)	β	95%CI	Exp (β)	β	95%CI	Exp (β)	β	95%CI
Plasma ferritin	1.025	0.011	-0.021 ; 0.042	1.052	0.022	0.009 ; 0.035	1.135	0.055	0.005 ; 0.105	1.197	0.078	0.027 ; 0.129	1.166	0.067	0.016; 0.117
Plasma ferritin quartiles															
Q1 (4.3-62.1 μg/L)	1	0		1	0		1	0		1	0		1	0	
Q2 (62.2-96.3 μg/L)	1.037	0.016	-0.080 ; 0.111	1.000	0.000	-0.040 ; 0.040	0.911	-0.041	-0.175 ; 0.096	1.020	0.009	-0.129 ; 0.148	1.022	0.010	-0.127 ; 0.147
Q3 (96.4-140.9 μg/L)	0.980	-0.009	-0.105 ; 0.088	1.036	0.015	-0.025 ; 0.055	0.966	-0.015	-0.154 ; 0.125	1.082	0.034	-0.108 ; 0.178	0.999	0.000	-0.141 ; 0.142
Q4 (141.0-250.0 μg/L)	1.146	0.059	-0.045 ; 0.164	1.150	0.061	0.017 ; 0.104	1.468	0.167	0.000 ; 0.333*	1.816	0.259	0.088 ; 0.429	1.747	0.242	0.073 ; 0.410
Plasma iron	0.863	-0.064	-0.199 ; 0.072	0.966	-0.015	-0.072 ; 0.042	0.631	-0.200	-0.399 ; -0.001	0.734	-0.134	-0.341 ; 0.074	0.641	-0.193	-0.394 ; 0.010
Plasma iron quartiles															
Q1 (0.12-0.59 μg/mL)	1	0		1	0		1	0		1	0		1	0	
Q2 (0.59-0.81 μg/mL)	0.925	-0.034	-0.134 ; 0.066	0.971	-0.013	-0.055 ; 0.029	0.801	-0.097	-0.248 ; 0.053	0.714	-0.146	-0.303 ; 0.008	0.738	-0.132	-0.284 ; 0.019
Q3 (0.81-1.06 μg/mL)	1.057	0.024	-0.091 ; 0.138	0.986	-0.006	-0.054 ; 0.042	0.671	-0.173	-0.352 ; 0.004	0.664	-0.178	-0.362 ; 0.006	0.614	-0.212	-0.391 ; -0.032
Q4 (1.06-1.91 μg/mL)	0.916	-0.038	-0.158 ; 0.082	0.977	-0.010	-0.061 ; 0.042	0.659	-0.181	-0.358 ; -0.005	0.659	-0.181	-0.363 ; 0.002	0.622	-0.206	-0.385 ; -0.027

Results were derived from linear mixed-effect models, using a random intercept for individuals and antibody or NAb levels as outcomes; models were adjusted for antibody level at baseline, vaccine type and number of vaccine doses received, time point of study visit, re-exposure at study visit, CRP, AGP, RBP, age, sex, smoking status, comorbidities. Models including plasma iron were further adjusted for time of the day of study visit.

Coefficients β are on the log10 scale; exp(β), i.e. 10^{β}, represents the multiplicative factor changes in antibody or NAb levels associated with a 50 μg/mL increase in plasma ferritin levels or a 1 μg/mL increase in plasma iron levels in continuous analyses, or compared to plasma ferritin or plasma iron Q1 in categorical analyses

Comorbidities included hypertension, diabetes, cardiovascular disease, respiratory disease, chronic kidney disease, cancer, and immunosuppression

Models were based on a study sample of n=563 for Anti-S IgA, n=563 for Anti-S IgG, n=212 for Anti-Ancestral NAb, n=212 for Anti-Delta NAb, n=212 for Anti-Omicron NAb for plasma ferritin and on a study sample of n=553 for Anti-S IgA, n=553 for Anti-S IgG, n=209 for Anti-Ancestral NAb, n=209 for Anti-Delta NAb, n=209 for Anti-Omicron NAb for plasma iron

*95%CI=-0.0003;0.3333

AGP: alpha(1)-acid glycoprotein; CI: confidence intervals; CRP: c-reactive protein; NAb: neutralizing antibodies; Q: quartile; RBP: retinol binding protein

Table S8: Longitudinal associations between plasma ferritin or plasma iron levels prior to vaccination and different immunity markers over 6 months, excluding participants with inflammation

	Anti-S IgA			Anti-S IgG			Anti-Ancestral NAb			Anti-Delta NAb			Anti-Omicron NAb		
	Exp (β)	β	95%CI	Exp (β)	β	95%CI	Exp (β)	β	95%CI	Exp (β)	β	95%CI	Exp (β)	β	95%CI
Plasma ferritin	1.037	0.016	-0.017 ; 0.048	1.053	0.023	0.009 ; 0.036	1.137	0.056	0.006 ; 0.106	1.198	0.079	0.028 ; 0.130	1.168	0.067	0.017 ; 0.118
Plasma ferritin quartiles															
Q1 (4.3-62.1 μg/L)	1	0		1	0		1	0		1	0		1	0	
Q2 (62.2-96.3 μg/L)	1.026	0.011	-0.086 ; 0.108	1.001	0.000	-0.040 ; 0.041	0.897	-0.047	-0.183 ; 0.090	1.016	0.007	-0.132 ; 0.147	1.014	0.006	-0.131 ; 0.144
Q3 (96.4-140.9 μg/L)	0.981	-0.008	-0.105 ; 0.088	1.030	0.013	-0.027 ; 0.053	0.970	-0.013	-0.153 ; 0.127	1.085	0.035	-0.107 ; 0.179	1.003	0.001	-0.139 ; 0.144
Q4 (141.0-250.0 μg/L)	1.173	0.069	-0.036 ; 0.176	1.157	0.063	0.019 ; 0.107	1.469	0.167	0.000 ; 0.334*	1.822	0.261	0.090 ; 0.431	1.750	0.243	0.074 ; 0.411
Plasma iron	0.852	-0.070	-0.207 ; 0.067	0.953	-0.021	-0.078 ; 0.037	0.635	-0.197	-0.396 ; 0.003	0.736	-0.133	-0.340 ; 0.076	0.646	-0.190	-0.392 ; 0.014
Plasma iron quartiles															
Q1 (0.12-0.59 μg/mL)	1	0		1	0		1	0		1	0		1	0	
Q2 (0.59-0.81 μg/mL)	0.942	-0.026	-0.127 ; 0.074	0.974	-0.012	-0.054 ; 0.031	0.800	-0.097	-0.249 ; 0.053	0.719	-0.144	-0.301 ; 0.012	0.740	-0.131	-0.284 ; 0.021
Q3 (0.81-1.06 μg/mL)	1.082	0.034	-0.081 ; 0.150	0.983	-0.008	-0.056 ; 0.041	0.675	-0.171	-0.350 ; 0.007	0.665	-0.177	-0.362 ; 0.008	0.617	-0.210	-0.389 ; -0.029
Q4 (1.06-1.91 μg/mL)	0.914	-0.039	-0.161 ; 0.082	0.968	-0.014	-0.065 ; 0.037	0.662	-0.179	-0.356 ; -0.002	0.664	-0.178	-0.361 ; 0.006	0.626	-0.204	-0.382 ; -0.024

Results were derived from linear mixed-effect models, using a random intercept for individuals and antibody or NAb levels as outcome; models were adjusted for antibody level at baseline, vaccine type and number of vaccine doses received, time point of study visit, re-exposure at study visit, CRP, AGP, RBP, age, sex, smoking status. Models including plasma iron were further adjusted for time of the day of study visit.
Coefficients β are on the log10 scale; exp(β), i.e. 10^{β}, represents the multiplicative factor changes in antibody or NAb levels associated with a 50 μg/mL increase in plasma ferritin levels or a 1 μg/mL increase in plasma iron levels in continuous analyses, or compared to plasma ferritin or plasma iron Q1 in categorical analyses
Inflammation was defined as CRP ≥ 5 mg/L or AGP ≥ 1 g/L
Models were based on a study sample of n=550 for Anti-S IgA, n=550 for Anti-S IgG, n=210 for Anti-Ancestral NAb, n=210 for Anti-Delta NAb, n=210 for Anti-Omicron NAb for plasma ferritin and on a study sample of n=540 for Anti-S IgA, n=540 for Anti-S IgG, n=207 for Anti-Ancestral NAb, n=207 for Anti-Delta NAb, n=207 for Anti-Omicron NAb for plasma iron
*95%CI=-0.00003;0.33363
AGP: alpha(1)-acid glycoprotein; CI: confidence intervals; CRP: c-reactive protein; NAb: neutralizing antibodies; Q: quartile; RBP: retinol binding protein

Table S9: Longitudinal associations between plasma ferritin or plasma iron levels prior to vaccination and different immunity markers over 6 months, not adjusting linear mixed-effect models for CRP and AGP

	**Anti-S IgA**			**Anti-S IgG**			**Anti-Ancestral NAb**			**Anti-Delta NAb**			**Anti-Omicron NAb**		
	Exp (β)	β	95%CI	Exp (β)	β	95%CI	Exp (β)	β	95%CI	Exp (β)	β	95%CI	Exp (β)	β	95%CI
Plasma ferritin	1.025	0.011	-0.021 ; 0.042	1.049	0.021	0.007 ; 0.034	1.137	0.056	0.006 ; 0.105	1.202	0.080	0.029 ; 0.130	1.171	0.069	0.018 ; 0.119
Plasma ferritin quartiles															
Q1 (4.3-62.1 μg/L)	1	0		1	0		1	0		1	0		1	0	
Q2 (62.2-96.3 μg/L)	1.026	0.011	-0.084 ; 0.106	0.995	-0.002	-0.042 ; 0.038	0.928	-0.032	-0.165 ; 0.101	1.042	0.018	-0.117 ; 0.154	1.046	0.020	-0.114 ; 0.154
Q3 (96.4-140.9 μg/L)	0.981	-0.009	-0.105 ; 0.088	1.031	0.013	-0.027 ; 0.054	0.982	-0.008	-0.146 ; 0.131	1.103	0.043	-0.098 ; 0.184	1.019	0.008	-0.131 ; 0.149
Q4 (141.0-250.0 μg/L)	1.148	0.060	-0.044 ; 0.164	1.139	0.056	0.013 ; 0.100	1.485	0.172	0.006 ; 0.337	1.850	0.267	0.098 ; 0.436	1.778	0.250	0.082 ; 0.417
Plasma iron	0.860	-0.065	-0.197 ; 0.066	0.983	-0.007	-0.062 ; 0.048	0.640	-0.194	-0.383 ; -0.004	0.722	-0.141	-0.338 ; 0.057	0.644	-0.191	-0.383 ; 0.002
Plasma iron quartiles															
Q1 (0.12-0.59 μg/mL)	1	0		1	0		1	0		1	0		1	0	
Q2 (0.59-0.81 μg/mL)	0.916	-0.038	-0.136 ; 0.059	0.981	-0.008	-0.049 ; 0.032	0.802	-0.096	-0.245 ; 0.051	0.714	-0.146	-0.300 ; 0.006	0.741	-0.130	-0.280 ; 0.019
Q3 (0.81-1.06 μg/mL)	1.054	0.023	-0.088 ; 0.133	1.000	0.000	-0.046 ; 0.047	0.669	-0.175	-0.349 ; -0.001	0.660	-0.181	-0.361 ; 0.000	0.615	-0.211	-0.387 ; -0.035
Q4 (1.06-1.91 μg/mL)	0.904	-0.044	-0.160 ; 0.072	0.992	-0.003	-0.052 ; 0.046	0.665	-0.177	-0.347 ; -0.008	0.658	-0.182	-0.357 ; -0.006	0.627	-0.202	-0.374 ; -0.031

Results were derived from linear mixed-effect models, using a random intercept for individuals and antibody or NAb levels as outcomes; models were adjusted for antibody level at baseline, vaccine type and number of vaccine doses received, time point of study visit, re-exposure at study visit, RBP, age, sex, smoking status. Models including plasma iron were further adjusted for time of the day of study visit.

Coefficients β are on the log10 scale; exp(β), i.e. 10^{β}, represents the multiplicative factor changes in antibody or NAb levels associated with a 50 μg/mL increase in plasma ferritin levels or a 1 μg/mL increase in plasma iron levels in continuous analyses, or compared to plasma ferritin or plasma iron Q1 in categorical analyses

Models were based on a study sample of n=563 for Anti-S IgA, n=563 for Anti-S IgG, n=212 for Anti-Ancestral NAb, n=212 for Anti-Delta NAb, n=212 for Anti-Omicron NAb for plasma ferritin and on a study sample of n=553 for Anti-S IgA, n=553 for Anti-S IgG, n=209 for Anti-Ancestral NAb, n=209 for Anti-Delta NAb, n=209 for Anti-Omicron NAb for plasma iron

*95%CI=-0.3609;-0.0004

AGP: alpha(1)-acid glycoprotein; CI: confidence intervals; CRP: c-reactive protein; NAb: neutralizing antibodies; Q: quartile; RBP: retinol binding protein

Table S10: Longitudinal associations between plasma ferritin or plasma iron levels prior to vaccination and neutralizing antibody levels over 6 months, adjusting linear mixed-effect models for seropositivity at baseline and knowledge of a prior infection

	Anti-Ancestral NAb			Anti-Delta NAb			Anti-Omicron NAb		
	Exp (β)	β	95%CI	Exp (β)	β	95%CI	Exp (β)	β	95%CI
Plasma ferritin	1.144	0.058	0.003 ; 0.114	1.209	0.082	0.026 ; 0.138	1.179	0.071	0.014 ; 0.128
Plasma ferritin quartiles									
Q1 (4.3-62.1 μg/L)	1	0		1	0		1	0	
Q2 (62.2-96.3 μg/L)	0.918	-0.037	-0.186 ; 0.113	1.026	0.011	-0.139 ; 0.163	1.031	0.013	-0.140 ; 0.168
Q3 (96.4-140.9 μg/L)	0.950	-0.022	-0.176 ; 0.133	1.069	0.029	-0.126 ; 0.186	0.981	-0.008	-0.166 ; 0.152
Q4 (141.0-250.0 μg/L)	1.605	0.205	0.021 ; 0.389	1.991	0.299	0.113 ; 0.485	1.938	0.287	0.098 ; 0.476
Plasma iron	0.474	-0.325	-0.539 ; -0.109	0.551	-0.259	-0.479 ; -0.036	0.466	-0.332	-0.552 ; -0.110
Plasma iron quartiles									
Q1 (0.12-0.59 μg/mL)	1	0		1	0		1	0	
Q2 (0.59-0.81 μg/mL)	0.787	-0.104	-0.270 ; 0.060	0.705	-0.152	-0.322 ; 0.016	0.724	-0.140	-0.309 ; 0.028
Q3 (0.81-1.06 μg/mL)	0.650	-0.187	-0.383 ; 0.009	0.646	-0.190	-0.390 ; 0.011	0.591	-0.228	-0.428 ; -0.028
Q4 (1.06-1.91 μg/mL)	0.545	-0.264	-0.457 ; -0.070	0.547	-0.262	-0.459 ; -0.064	0.502	-0.299	-0.496 ; -0.102

Results were derived from linear mixed-effect models, using a random intercept for individuals and NAb levels as outcome; models were adjusted for seropositivity at baseline and knowledge of a prior infection, vaccine type and number of vaccine doses received, time point of study visit, re-exposure at study visit, CRP, AGP, RBP, age, sex, smoking status. Models including plasma iron were further adjusted for time of the day of study visit.

Coefficients β are on the log10 scale; exp(β), i.e. 10^{β}, represents the multiplicative factor changes in NAb levels associated with a 50 μg/mL increase in plasma ferritin levels or a 1 μg/mL increase in plasma iron levels in continuous analyses, or compared to plasma ferritin or plasma iron Q1 in categorical analyses

Models were based on a study sample of n=212 for Anti-Ancestral NAb, n=212 for Anti-Delta NAb, n=212 for Anti-Omicron NAb for plasma ferritin and on a study sample of n=209 for Anti-Ancestral NAb, n=209 for Anti-Delta NAb, n=209 for Anti-Omicron NAb for plasma iron

AGP: alpha(1)-acid glycoprotein; CI: confidence intervals; CRP: c-reactive protein; NAb: neutralizing antibodies; Q: quartile; RBP: retinol binding protein

Table S11: Antibody and neutralizing antibody levels in the overall study population and by plasma ferritin and plasma iron quartiles at 6 months

	**Anti-S IgA (MFI ratio)** Geometric mean (95% CI)	**Anti-S IgG (MFI ratio)** Geometric mean (95% CI)	**Anti-Ancestral NAb (IC_{50})** Geometric mean (95% CI)	**Anti-Delta NAb (IC_{50})** Geometric mean (95% CI)	**Anti-Omicron NAb (IC_{50})** Geometric mean (95% CI)
**Overall**	14.5 (12.7-16.6)	39.8 (37.1-42.8)	71.8 (60.2-85.7)	41.5 (34.2-50.5)	29.4 (22.0-39.2)
**Ferritin quartiles**					
Q1 (4.3-62.1 μg/L)	12.9 (10.2-16.2)	43.9 (38.2-50.4)	71.3 (51.5-98.7)	39.8 (30.1-52.5)	25.0 (16.9-36.9)
Q2 (62.2-96.3 μg/L)	15.6 (12.2-19.9)	41.7 (36.5-47.6)	65.6 (48.7-88.5)	48.2 (33.7-69.0)	27.7 (16.0-47.9)
Q3 (96.4-140.9 μg/L)	13.2 (9.5-18.3)	36.4 (30.9-43.0)	81.8 (58.7-113.9)	38.1 (25.9-55.9)	24.6 (10.1-60.0)
Q4 (141.0-250.0 μg/L)	17.0 (12.7-22.8)	37.2 (32.2-43.1)	72.5 (36.6-143.9)	41.7 (15.1-114.8)	53.5 (9.6-299.3)
**Plasma iron quartiles**					
Q1 (0.12-0.59 μg/mL)	14.4 (11.0-18.8)	43.4 (37.7-49.8)	89.4 (63.8-125.1)	47.7 (31.7-71.6)	43.8 (19.0-100.8)
Q2 (0.59-0.81 μg/mL)	13.0 (9.9-17.1)	37.1 (31.7-43.4)	53.8 (33.4-86.7)	34.4 (20.4-58.0)	21.5 (10.6-43.9)
Q3 (0.81-1.06 μg/mL)	17.4 (13.1-23.0)	38.4 (33.1-44.6)	68.1 (50.3-92.2)	38.2 (26.6-54.8)	20.8 (11.8-36.5)
Q4 (1.06-1.91 μg/mL)	13.1 (10.0-17.2)	39.8 (34.5-45.8)	76.5 (54.1-108.2)	44.3 (30.3-64.8)	30.0 (19.8-45.5)

Seropositivity was defined as MFI ratio > 6.5 for IgA, MFI ratio > 6.0 for IgG antibodies; neutralizing capacity was defined as serum dilution IC_{50} > 50 for Anti-Ancestral, Anti-Delta and Anti-Omicron NAb

Plasma ferritin: based on a study sample of n=278 for Anti-S IgA, n=205 for Anti-S IgG, n=101 for Anti-Ancestral NAb, n=73 for Anti-delta NAb, n=23 for Anti-omicron NAb

Plasma iron: based on a study sample of n=273 for Anti-S IgA, n=203 for Anti-S IgG, n=101 for Anti-Ancestral NAb, n=73 for Anti-delta NAb, n=23 for Anti-omicron NAb

CI: confidence interval; IC_{50}: half maximal inhibitory concentration serum dilution; MFI: mean fluorescence intensity; NAb: neutralizing antibodies; Q: quartile

Table S12: Seropositivity and neutralizing capacity at 6 months in the overall study population and by plasma ferritin and plasma iron quartiles

	Anti-S IgA<br>Seropositivity (%)	Anti-S IgG<br>Seropositivity (%)	Anti-Ancestral NAb<br>Neutralizing capacity (%)	Anti-Delta NAb<br>Neutralizing capacity (%)	Anti-Omicron NAb<br>Neutralizing capacity (%)
**Overall**	75.9	99.0	66.3	41.1	21.7
**Ferritin quartiles**					
Q1 (4.3-62.1 μg/L)	74.1	100.0	71.0	37.0	12.5
Q2 (62.2-96.3 μg/L)	80.3	98.4	65.6	38.9	28.6
Q3 (96.4-140.9 μg/L)	65.7	98.0	65.2	38.9	25.0
Q4 (141.0-250.0 μg/L)	83.1	100.0	60.0	60.0	25.0
**Plasma iron quartiles**					
Q1 (0.12-0.59 μg/mL)	76.1	98.2	77.8	31.8	42.9
Q2 (0.59-0.81 μg/mL)	71.2	100.0	54.5	33.3	0.0
Q3 (0.81-1.06 μg/mL)	84.1	100.0	58.3	41.2	0.0
Q4 (1.06-1.91 μg/mL)	73.1	98.0	71.4	57.9	28.6

Seropositivity was defined as MFI ratio > 6.5 for IgA and MFI ratio > 6.0 for IgG antibodies; neutralizing capacity was defined as serum dilution IC_{50} > 50 for Anti-Ancestral, Anti-Delta and Anti-Omicron NAb

Plasma ferritin: based on a study sample of n=278 for Anti-S IgA, n=205 for Anti-S IgG, n=101 for Anti-Ancestral NAb, n=73 for Anti-delta NAb, n=23 for Anti-omicron NAb

Plasma iron: based on a study sample of n=273 for Anti-S IgA, n=203 for Anti-S IgG, n=101 for Anti-Ancestral NAb, n=73 for Anti-delta NAb, n=23 for Anti-omicron NAb

IC_{50}: half maximal inhibitory concentration serum dilution; MFI: mean fluorescence intensity; NAb: neutralizing antibodies; Q: quartile

Table S13: Longitudinal association between plasma iron levels prior to vaccination and neutralizing antibody levels over 6 months, not adjusting linear mixed-effect models for baseline antibody levels

	**Anti-Ancestral NAb**			**Anti-Delta NAb**			**Anti-Omicron NAb**		
	Exp (β)	β	95%CI	Exp (β)	β	95%CI	Exp (β)	β	95%CI
Plasma iron	0.411	-0.386	-0.620 ; -0.151	0.474	-0.324	-0.565 ; -0.080	0.394	-0.405	-0.649 ; -0.158
Plasma iron quartiles									
Q1 (0.12-0.59 μg/mL)	1	0		1	0		1	0	
Q2 (0.59-0.81 μg/mL)	0.724	-0.140	-0.321 ; 0.040	0.647	-0.189	-0.375 ; -0.004	0.658	-0.182	-0.370 ; 0.006
Q3 (0.81-1.06 μg/mL)	0.565	-0.248	-0.462 ; -0.034	0.557	-0.254	-0.473 ; -0.034	0.502	-0.300	-0.521 ; -0.076
Q4 (1.06-1.91 μg/mL)	0.493	-0.307	-0.518 ; -0.096	0.493	-0.307	-0.523 ; -0.090	0.445	-0.352	-0.570 ; -0.131

Results were derived from linear mixed-effect models, using a random intercept for individuals and NAb levels as outcomes; models were adjusted for vaccine type and number of vaccine doses received, time point of study visit, re-exposure at study visit, time of the day of study visit, CRP, AGP, RBP, age, sex, smoking status.

Coefficients β are on the log10 scale; exp(β), i.e. 10^{β}, represents the multiplicative factor changes in NAb levels associated with a 1 μg/mL increase in plasma iron levels in continuous analyses, or compared to plasma iron Q1 in categorical analyses

Models were based on a study sample of n=209

AGP: alpha(1)-acid glycoprotein; CI: confidence intervals; CRP: c-reactive protein; NAb: neutralizing antibodies; Q: quartile; RBP: retinol binding protein
